# Supplementary material for: Could social prescribing contribute to type 2 diabetes prevention in people at high risk? Protocol for a realist, multilevel, mixed methods review and evaluation
Source: BMJ Open. 2021 Apr 9;11(4):e042303. doi: 10.1136/bmjopen-2020-042303 (PMC8043019; doi:10.1136/bmjopen-2020-042303)
Supplement: Supplementary data [file bmjopen-2020-042303supp001.pdf]

Supplementary File 1. Literature searches

The final search strategy included combinations of search terms for the concepts 'social prescribing', 'primary care', 'community referral', 'diabetes', and was informed by previous related reviews on SP (1–3) and further developed in MEDLINE (Ovid) using an iterative process of adding, removing and refining search terms.

In September 2019, the following databases were searched: MEDLINE (Ovid), EMBASE (Ovid), PsycINFO (Ovid), Scopus (Elsevier), Web of Science (Clarivate Analytics), CINAHL Plus (EBSCO), PubMed (NCBI), International Bibliography of the Social Sciences - IBSS (ProQuest), The Cochrane Database of Systematic Reviews (The Cochrane Library), Campbell Collaboration, Open Grey (INISR-CNRS), King's Fund Database and LILACS (BIREME).

Database alerts were set up to identify studies published between October 2019 and March 2020.

Database: MEDLINE

Host: Ovid.

Date searched: 27 September 2019.

Searcher: Sara Calderón, Yasmin Milner.

Hits: 129.

Strategy:

1. (social prescri* and type 2 diabetes).mp.
2. (social prescri* or community referral).mp.
3. (social prescri* or community referral or non medical referral).mp.
4. (social prescri* and primary health care).mp.
5. (social prescri* and primary care).mp.
6. (social prescri* or community referral or referral scheme).mp.
7. ((((social prescri* or community referral) and primary care) or primary health care) and diabetes).mp.
8. ((social prescri* or community referral) and primary health care and diabetes).mp.
9. 1 or 2 or 3 or 4 or 5 or 8

Database: EMBASE

Host: Ovid.

Date searched: 27 September 2019.

Searcher: Sara Calderón, Yasmin Milner.

Hits: 271.

Strategy:

1. (social prescri* and type 2 diabetes).mp.
2. (social prescri* or community referral).mp.
3. (social prescri* or community referral or non medical referral).mp.
4. (social prescri* and primary health care).mp.
5. (social prescri* and primary care).mp.
6. (social prescri* or community referral or referral scheme).mp.
7. ((((social prescri* or community referral) and primary care) or primary health care) and diabetes).mp.
8. ((social prescri* or community referral) and primary health care and diabetes).mp.
9. 1 or 2 or 3 or 4 or 5 or 8

Database: PsycINFO

Host: Ovid.

Date searched: 27 September 2019.

Searcher: Sara Calderón, Yasmin Milner.

Hits: 113.

Strategy:

1. (social prescri* and type 2 diabetes).mp.
2. (social prescri* or community referral).mp.
3. (social prescri* or community referral or non medical referral).mp.
4. (social prescri* and primary health care).mp.
5. (social prescri* and primary care).mp.
6. (social prescri* or community referral or referral scheme).mp.
7. ((((social prescri* or community referral) and primary care) or primary health care) and diabetes).mp.
8. ((social prescri* or community referral) and primary health care and diabetes).mp.
9. 1 or 2 or 3 or 4 or 5 or 8

Database: Scopus

Host: Elsevier.

Date searched: 29 September 2019.

Searcher: Sara Calderón, Yasmin Milner.

Hits: 355.

Strategy:

1. TITLE-ABS-KEY ("Social Prescri*" OR "community referral" AND "type 2 diabetes")
2. TITLE-ABS-KEY ("Social Prescri*" OR "community referral")
3. TITLE-ABS-KEY ("Social Prescri*" OR "community referral" AND "diabetes" AND "primary health care")
4. TITLE-ABS-KEY ("Social Prescri*" OR "community referral" AND "primary health care")

5. TITLE-ABS-KEY ("Social Prescri*" AND "primary health care")

Database: CINAHL Plus

Host: EBSCO.

Date searched: 30 September 2019.

Searcher: Sara Calderón, Yasmin Milner.

Hits: 165.

Strategy:

1. "Social prescri*" OR "community referral"

Database: PubMed

Host: NCBI.

Date searched: 30 September 2019.

Searcher: Sara Calderón, Yasmin Milner.

Hits: 183.

Strategy:

1. (((((((social prescri*) OR "community referral")) AND "primary health care")) OR ((social prescri*) OR "community referral"))) OR ((((social prescri*) OR "community referral")) AND diabetes)
2. (((((social prescri*) OR "community referral")) AND "primary health care")) OR ((social prescri*) OR "community referral")

Database: Web of Science

Host: Clarivate Analytics.

Date searched: 30 September 2019.

Searcher: Sara Calderón, Yasmin Milner.

Hits: 183.

Strategy:

1. ("social prescri*")
2. ("social prescri*" OR "community referral")
3. ("social prescri*" OR "community referral") *AND* (diabetes)
4. ("social prescri*" OR "community referral") *AND* ("primary health care")
5. #4 OR #3 OR #2 OR #1

Database: IBSS

Host: ProQuest.

Date searched: 30 September 2019.

Searcher: Sara Calderón, Yasmin Milner.

Hits: 11.

Strategy:

1. noft("community referral") OR noft("social prescri*")

Database: Cochrane Library

Host: Cochrane Collaboration.

Date searched: 30 September 2019.

Searcher: Sara Calderón, Yasmin Milner.

Hits: 28.

Strategy:

1. "social prescri*" in Title Abstract Keyword OR "community referral" in Title Abstract Keyword AND "primary care" in Title Abstract Keyword
2. "social prescri*" in Title Abstract Keyword OR "community referral" in Title Abstract Keyword AND diabetes in Title Abstract Keyword
3. "social prescri*" in Title Abstract Keyword OR "community referral" in Title Abstract Keyword

Database: Open Grey

Host: INISR-CNRS.

Date searched: 30 September 2019.

Searcher: Sara Calderón, Yasmin Milner.

Hits: 1.

Strategy:

1. "social prescribing" OR "community referral"

Database: LILACS

Host: BIREME.

Date searched: 30 September 2019.

Searcher: Sara Calderón, Yasmin Milner.

Hits: 93.

Strategy:

1. (tw:("community referral"))
2. (tw:("community referral")) AND (tw:("social prescribing"))

Database: King's Fund

Host: The King's Fund.

Date searched: 3 October 2019.

Searcher: Sara Calderón, Yasmin Milner.

Hits: 218.

Strategy:

1. "social prescribing"
2. "community referral" AND "social prescribing"

REFERENCES:

1. Public Health England. Effectiveness of social prescribing - An evidence synthesis [Internet]. London; 2019 [cited 2019 Sep 29]. Available from: www.facebook.com/PublicHealthEngland

2. Pilkington K, Loef M, Polley M. Searching for Real-World Effectiveness of Health Care Innovations: Scoping Study of Social Prescribing for Diabetes. J Med Internet Res [Internet]. 2017 Feb 2 [cited 2018 May 14];19(2):e20–e20. Available from: http://www.ncbi.nlm.nih.gov/pubmed/28153817

3. Bickerdike L, Booth A, Wilson PM, Farley K, Wright K, Wilson PM. Social prescribing: less rhetoric and more reality. A systematic review of the evidence. BMJ Open [Internet]. 2017 [cited 2018 Jun 2];7(4):e013384. Available from: http://dx.doi.org/
