# Supplementary material for: Could social prescribing contribute to type 2 diabetes prevention in people at high risk? Protocol for a realist, multilevel, mixed methods review and evaluation
Source: BMJ Open. 2021 Apr 9;11(4):e042303. doi: 10.1136/bmjopen-2020-042303 (PMC8043019; doi:10.1136/bmjopen-2020-042303)
Supplement: Author's manuscript [file bmjopen-2020-042303.draft_revisions.pdf]

BMJ Open is committed to open peer review. As part of this commitment we make the peer review history of every article we publish publicly available.

When an article is published we post the peer reviewers' comments and the authors' responses online. We also post the versions of the paper that were used during peer review. These are the versions that the peer review comments apply to.

The versions of the paper that follow are the versions that were submitted during the peer review process. They are not the versions of record or the final published versions. They should not be cited or distributed as the published version of this manuscript.

BMJ Open is an open access journal and the full, final, typeset and author-corrected version of record of the manuscript is available on our site with no access controls, subscription charges or pay-per-view fees (http://bmjopen.bmj.com).

If you have any questions on BMJ Open's open peer review process please email info.bmjopen@bmj.com

# Could social prescribing contribute to type 2 diabetes prevention in populations at high risk? Protocol for a realist multi-level, mixed-methods review and evaluation.

| | |
|---|---|
| Journal: | *BMJ Open* |
| Manuscript ID | bmjopen-2020-042303 |
| Article Type: | Protocol |
| Date Submitted by the Author: | 02-Jul-2020 |
| Complete List of Authors: | Calderón Larrañaga, Sara; Queen Mary University of London,<br>Clinch , Megan; Queen Mary University of London<br>Greenhalgh, Trisha; University of Oxford, Nuffield Department of Primary Care Health Sciences<br>Finer, Sarah; Barts and the London School of Medicine and Dentistry, Queen Mary University of London, Blizard Institute |
| Keywords: | PRIMARY CARE, SOCIAL MEDICINE, DIABETES & ENDOCRINOLOGY |
| | |

*I, the Submitting Author has the right to grant and does grant on behalf of all authors of the Work (as defined in the below author licence), an exclusive licence and/or a non-exclusive licence for contributions from authors who are: i) UK Crown employees; ii) where BMJ has agreed a CC-BY licence shall apply, and/or iii) in accordance with the terms applicable for US Federal Government officers or employees acting as part of their official duties; on a worldwide, perpetual, irrevocable, royalty-free basis to BMJ Publishing Group Ltd ("BMJ") its licensees and where the relevant Journal is co-owned by BMJ to the co-owners of the Journal, to publish the Work in this journal and any other BMJ products and to exploit all rights, as set out in our licence.*

*The Submitting Author accepts and understands that any supply made under these terms is made by BMJ to the Submitting Author unless you are acting as an employee on behalf of your employer or a postgraduate student of an affiliated institution which is paying any applicable article publishing charge ("APC") for Open Access articles. Where the Submitting Author wishes to make the Work available on an Open Access basis (and intends to pay the relevant APC), the terms of reuse of such Open Access shall be governed by a Creative Commons licence – details of these licences and which Creative Commons licence will apply to this Work are set out in our licence referred to above.*

*Other than as permitted in any relevant BMJ Author's Self Archiving Policies, I confirm this Work has not been accepted for publication elsewhere, is not being considered for publication elsewhere and does not duplicate material already published. I confirm all authors consent to publication of this Work and authorise the granting of this licence.*

For peer review only

**TITLE:**

Could social prescribing contribute to type 2 diabetes prevention in populations at high risk? Protocol for a realist multi-level, mixed-methods review and evaluation.

**CORRESPONDING AUTHOR:** Sara Calderón-Larrañaga, s.calderon@qmul.ac.uk. Postal address: 58 Turner St, Whitechapel, London E1 2AB.

**AUTHORS**: Sara Calderón-Larrañaga (1,2), Megan Clinch (1), Trish Greenhalgh (3), Sarah Finer (1,4).

1. Centre for Primary Care and Mental Health, Institute of Population Health Sciences, Queen Mary University of London. London, UK
2. Bromley By Bow Health Partnership. London, UK
3. Nuffield Department of Primary Care Health Sciences, University of Oxford, Oxford, UK
4. Barts Health NHS Trust, London, UK

**WORD COUNT:** 3,611

**KEYWORDS:** social prescribing, type 2 diabetes, primary health care, realist methodology

For peer review only

**ABSTRACT:**

**Introduction.** Social Prescribing is an innovation being widely adopted within the UK National Health Service Policy as a way of addressing the social determinants of long-term conditions, such as type 2 diabetes (T2D). It generally involves linking patients in primary care with non-medical community-based interventions. Despite widespread national support, evidence for the effectiveness of social prescribing is both insufficient and contested. In this study, we will investigate whether social prescribing can contribute to T2D prevention and, if so, when, how and in what circumstances it might best be introduced.

**Methods and analysis.** We will draw on realist evaluation to investigate the complex interpersonal, organizational, social and policy context in which social prescribing relevant to T2D prevention is implemented. We will set up a stakeholders' group to advise us throughout the study, which will be conducted over 3 interconnected stages. In Stage 1, we will undertake a realist review to synthesize the current evidence base for social prescribing. In Stage 2, we will investigate how social prescribing relevant to people at high risk of T2D 'works' in a multi-ethnic, socio-economically diverse community and any interactions with existing T2D prevention services using qualitative, quantitative and realist methods. In Stage 3 and building on previous stages, we will synthesise a 'transferable framework' that will guide implementation and evaluation of social prescribing relevant to T2D prevention at scale.

**Ethics and Dissemination.** Research Ethics Committee (REC) approval has been granted (reference 20/LO/0713). This project will potentially inform the adaptation of social prescribing services to better meet the needs of people at high risk of T2D in socioeconomically deprived areas. Findings may also be transferable to other long-term conditions. Dissemination will be undertaken as a continuous process, supported by the stakeholder group. Tailored outputs will target the following audiences: (1) service providers and commissioners; (2) people at high risk of T2D and community stakeholders; and (3) policy and strategic decision makers.

For peer review only

**STRENGTHS AND LIMITATIONS OF THIS STUDY:**

- Our study contributes to a much-needed evidence base for both social prescribing (SP) and its real-world implementation in specific populations, such as those at high risk of type 2 diabetes (T2D)

- The realist methodology will enable to evaluate existing SP programmes within the wider relational, organisational and policy context where they are implemented and delivered

- We will seek participatory involvement from our stakeholders' advisory group throughout the study, which will strengthen the practical relevance and transferability of findings

- The study does not intend to generate an effect size, but findings may guide the implementation and evaluation of SP initiatives relevant to T2D prevention at scale

For peer review only

**INTRODUCTION:**

Social Prescribing (SP) is an innovation being widely adopted in the UK, including at policy-level within the National Health Service (NHS) 'Long Term Plan'(1). Although its definition varies, SP generally involves linking patients in primary care with community services offering employment, housing or financial advice, as well as a range of 'healthy lifestyle' activities, such as cooking classes, weight management or exercise programmes(2). Health and lifestyle issues are supposed to be addressed alongside socio-economic matters in an integrated way, usually with the help of a 'social prescriber' (also called 'link worker') who acts as link between health professionals and the wide range of community and voluntary services relevant to patients' situation(3,4). Activities are typically non-medicalised, provided locally and potentially more accessible and culturally appropriate to the local communities(5).

Proponents of SP suggest it may improve health outcomes and redress health inequalities by addressing patients' social and behavioural determinants of health(6). It is also argued that SP could improve the efficient use of NHS and social care resources by enhancing self-care and strengthened community networks(7). However, current evidence base for SP is both scarce and contested. A recent systematic review published by Public Health England comprising 8 UK studies (1 cluster RCT and 7 pre/post-test studies) found no clear evidence for effectiveness of SP schemes(8). In an earlier systematic review, Bickerdike and colleagues did not find enough evidence to prove either success or value for money across 15 studies(9). Further systematic reviews on SP have failed to report consistent health, service utilization or cost results(9-14).

Authors suggest that the evidence gap on SP may be, in part, due to inadequate methodological approaches to gathering, assessing and/or synthesising data(2). Because SP interventions have fuzzy boundaries, multiple and inter-related components and are extremely sensitive to the context, conventional research designs (namely randomised controlled trials (RCTs) and/or previous systematic reviews) may not be the most appropriate approach to evaluation(15,16). Although RCTs would enable to compare the effectiveness of different variations of SP programmes, the methodological requirements to do so could impede the analysis of the interplay between programme implementation, the individuals involved, the programme's context and the wider social environment(17). Novel, complexity-informed methodological approaches are therefore needed to understand 'why', 'for whom', and 'in what context' SP programs might (or might not) work(15,18).

For peer review only

There is also a need to evaluate the role of SP in specific patient groups and populations(19,20). This study will use type 2 diabetes (T2D) prevention as an exemplar and investigate the impact of SP in populations at high risk of the condition based on the following considerations. First, T2D is a major public health concern, associated with reduced quality of life and life expectancy, and considerable socioeconomic consequences(21). Although individual behavioural risk factors, such as physical inactivity and poor nutrition, are important determinants of T2D risk, these are heavily influenced and constrained by higher-level social and demographic factors(22,23), which appear to be major driving forces behind escalating T2D epidemics and health inequalities(24,25). For T2D prevention strategies to be effective at population level and in the most high-risk groups, they most likely will need to address these social determinants in addition to focusing purely on individual-level behavioural interventions(26). Second, current NHS delivered T2D prevention programmes, such us ‘Healthier You’(27), have not demonstrated high uptake(28). The Healthier You intervention consists of 18 educational sessions across 9 months offered to people at high risk of T2D, but its effectiveness in preventing the condition remains uncertain(29). Additionally, a social gradient exists in those engaging with, and gaining benefit from the Healthier You programme(30). There is significant concern that low uptake and high attrition may, at least in part, reflect the fact that individualised behavioural interventions do not adequately address critical social determinants of T2D(31, 32). Evaluating the impact of SP on a specific health area, such as T2D, will potentially enable to examine whether and how such interventions may be able to complement existing individualised behavioural approaches.

**Setting and context:**

This study will be based in Tower Hamlets, a multi-ethnic inner-city district in the East End of London, UK, where overall and T2D-related health outcomes have proved to be poorer compared to the national average(33) despite high quality care(34). Tower Hamlets is one of the most deprived boroughs in the UK and is characterised by its great ethnic diversity, with over half of all residents having a minority ethnic background(35).

Local health intelligence data suggests that age-adjusted deaths directly attributable to diabetes are 8.93 per 100,000, compared to 5.06 across London and 5.06 across England(34). Rates of T2D in Tower Hamlets are significantly higher amongst sub-populations of lowest socio-economic status and South-Asian population(33,35,36). For instance, South Asians have a risk of developing T2D that is two-fold greater than the local white population(37) and suffer from faster chronic kidney progression when they have diabetes(38). Tower Hamlets has become one

For peer review only

of the 27 areas across the country chosen to be part of the first wave rollout of the national NHS Diabetes Prevention Programme, ‘Healthier You’.

Tower Hamlets is also at the forefront of another innovative service model, SP, that is being rapidly adopted throughout the NHS. Tower Hamlets has a long-established local voluntary and community sector (VCS)(36) who have a long history of delivering health promotion activities, including those relevant to T2D prevention. SP is being rolled out borough-wide by the Clinical Commissioning Group, aimed at strengthening and expanding the partnership between the health and voluntary sectors to all local Primary Health Care centres, building on the structure and experience developed in the pre-existing VCS and SP schemes(40). Each of the borough’s 37 primary care settings is linked to at list one ‘social prescriber’(40).

The proposed research will examine the existing evidence base for SP, and critically evaluate SP implementation and delivery in Tower Hamlets, using the following specific aims and questions:

**Aims:**

1. To investigate the possible impact of SP on populations at high risk of T2D.
2. To inform the design, implementation and evaluation of SP initiatives relevant to people at high risk of T2D.

**Research questions:**

1. To what extent might SP meet the complex health and social needs of socio-economically diverse populations at high risk of T2D?
2. What are the main conditions and mechanisms (‘active ingredients’) that might explain the success or failure of SP programmes relevant to people at risk of T2D?
3. How do existing SP programmes address questions of implementation and evaluation, and what are the knowledge gaps critical to understanding their success or failure within a health system?

**METHODS:**

**Theoretical and conceptual framework:**

We will draw on realism, a theory-driven methodology that seeks to facilitate deep understanding of how complex interventions, such as SP, work and in what circumstances(41,

For peer review only

42). A central aspect of realism is its analysis of causation, which rejects the standard Humean model of regular succession of events. Realism takes the view that ‘what causes something to happen has nothing to do with the number of times we observe it happening, instead identifying causal (also called ‘generative’) mechanisms of action and explaining how they work, whether they have been activated, and if so under what conditions’(41). Our task as researchers will be to untangle the ‘web of causation’(43) identified using this realist approach, and to critically illuminate the underlying structures and mechanisms that might shape and generate empirically observed outcomes.

In order to explore these underlying assumptions, realist methodology proposes the identification of context-mechanism-outcome configurations (CMOc)(44). A CMOc is a hypothesis that the program works (or does not work) (O) because of the action of some underlying mechanisms (M), which only comes into operation in particular contexts (C). ‘*If* the right processes operate in the right conditions *then* the program will prevail’(45). These kind of theoretical explanations, sufficiently abstract to draw transferable lessons but closed enough to the observable data to enable empirical testing and refinement, are referred to as ‘programme theories’(46).

CMOc may link to each other over time, with the outcome of one phase becoming the context of the next configuration in the chain of implementation steps (known as ‘ripple effect’)(47). Yet, CMOc may also organize concentrically(45). In this study, we propose a layered and dynamic realist framework (Figure 1) to investigate the complex interpersonal, organisational, social and policy context in which SP relevant T2D is implemented, and illuminate key domains and tensions at these different and interconnected levels. A theory-testing strategy will be used to unpack these causal mechanisms and interrelated CMOc. Preliminary theoretical explanations will be made explicit and iteratively tested, confronted and refined using relevant and varied evidence from the primary literature (realist review(48)) and/or empirical data (realist evaluation(49)).

**Research Plan:**

The study will be conducted in three interlinked stages over three years as follows:

- Stage 1 – Development and refinement of a realist programme theory for SP interventions relevant to populations at high risk of T2D using a critical literature review (realist synthesis).

For peer review only

- Stage 2 – Empirical testing and further refinement of a realist programme theory (guided by Stage 1) in a socio-economically deprived, ethnically-diverse community at high risk of T2D using qualitative, quantitative and realist methods (realist evaluation).

- Stage 3 – Synthesis of secondary (Stage 1) and primary (Stage 2) evidence, involving the development of a transferable framework for guiding implementation and evaluation of SP relevant to T2D prevention at scale.

In parallel and as represented in Figure 2, we will set up a stakeholders' group comprising representatives of the VCS, primary care leaders, members of Public Health England and relevant national charitable organisations (such as SP Network, Deep End Group, and/or the National Academy for Social Prescribing). Via individual and group interviews, the study objectives and ongoing research outcomes will be iteratively shared, leading to discussions that will seek to explore and highlight stakeholders' opinions and concerns about SP, T2D prevention and the emerging programme theory.

**Stage 1. Development and refinement of a realist programme theory (Realist Synthesis)**

Following the Realist And Meta-narrative Evidence Syntheses: Evolving Standards (RAMESES)(48,50), we will undertake a realist review in four iterative stages: (A) Searching for evidence, (B) Selecting and appraising literature, (C) Extracting and organizing data and (D) Synthesizing the evidence and drawing conclusions(51).

(A) Searching for evidence

Searches will be organised and performed in two phases. First, we will perform a 'theory mapping exercise' to identify the rationales for why and how SP might (or might not) play a role in T2D prevention and build preliminary programme theories that will be negotiated within the research and stakeholders' groups. Second, we will undertake targeted searches aimed at refining these candidate programme theories.

We will use free-text and indexing search terms employed in previous systematic reviews(8,9, 19). Terms for the second search will depend on the results from the first phase and will be discussed with a specialist librarian and the review team. We will search for qualitative, quantitative and mixed-methods studies in relevant electronic databases and grey literature resources (specified in Appendix 1). We will also undertake forwards and backwards citation chasing on included studies, hand-searching of key journals, and study author contact where

For peer review only

appropriate(52). Results will be exported to Rayyan QCRI for de-deduplication. The searching process will be reproduced by a second reviewer for consistency.

(B) Selecting and appraising articles

Studies will be included if they consider adults (18+) in primary care settings and focus on interventions that link patients in primary care with community-based resources. Studies will be excluded if written in languages other than English, Spanish or French. Inclusion and exclusion criteria will continue to be refined throughout the review as theoretical understanding develops, and until theoretical saturation has been reached. Literature will be appraised based on their relevance at the point of inclusion ('does it contribute to theory building and testing?') and rigour ('are the methods used to generate the relevant data credible and trustworthy?')(50). A 10% random subsample of screening decisions will be reviewed by a second reviewer for consistency.

(C) Extracting and organizing data

The main reviewer will read all included articles, extract descriptive study characteristics and carry out manual coding. Full texts and the coding framework resulting from the manual analysis will then be uploaded into NVivo 11 (qualitative data management software). Initial coding will comprise broad conceptual categories that will progressively be refined both inductively and deductively, and reframed applying a realist logic of analysis. This means that we will work iteratively to identify sections of the text related to contexts (C), mechanisms (M) and outcomes (O) and organize data in level-specific CMOc(51). Again, a 10% random subsample of coded articles will be reviewed by a second reviewer for consistency, and disagreements will be solved by discussion.

(D) Synthesizing the evidence and drawing conclusions

We will use evidence synthesis processes proposed by realist methodology (e.g. juxtaposition of data sources, reconciling 'contradictory' or disconfirming data, consolidation of sources of evidence)(45) to articulate clear and contextualised explanations on how and why certain patterns related to SP relevant to T2D prevention occur. These theory-informed, evidence-based, multilevel propositions will guide the empirical realist evaluation and the development of a transferable framework in stages 2 and 3, respectively.

For peer review only

**Stage 2. Empirical testing and further refinement of a realist programme theory (Realist Evaluation)**

(A) Study Design

We will undertake a realist, multi-level and mixed-methods evaluation of an existing SP programme, based in Tower Hamlets, east London (the setting has been described above). The appropriateness and relevance of these approaches in the study and evaluation of complex, multi-dimensional, dynamic phenomena (such as SP) have been well stablished in the literature(53-55).

At microlevel, we will characterise SP users and investigate their experience throughout the SP pathway (from initial referral to subsequent assessment by the link worker and unfolding community-based activities), paying special attention to the dynamic interaction between the different actors involved (patients, primary care workers, link workers, members of local community organisations, local authority/public health services). At mesolevel, we will explore how a primary care service and the voluntary and community sector serving a community at high risk of T2D interact and operate to enable SP. We will also study the potential interaction of SP schemes with existing NHS T2D prevention programmes, such us 'Healthier You'. At macrolevel, we will investigate the barriers, facilitators and incentives to supporting SP relevant to T2D within the managerial and political sphere, and explore the interests and significances of SP for policymakers and other key stakeholders. Following a convergent mixed-methods study design, qualitative and quantitative data will be collected and analysed 'in parallel' (during a similar timeframe) using an interactive approach, where data collection and analysis might drive changes in ongoing data collection procedures(56,57).

(B) Data collection

Data will be generated through a number of qualitative and quantitative methods (Table 1):

- Individual and group interviews. We will purposively select SP users living in an area at high risk of T2D (including those who did not turn up for appointments or declined to engage with the activities), members of VCS organisations involved in SP, as well as local primary care workers, social prescribers and policy makers until a sufficient sample has been obtained. Sampling will be guided by the strategies specified in table 1 and the findings of the realist review, as to the characteristics and circumstances that play a role in the success or failure of SP. An initial invitation letter and consent form will be

For peer review only

provided and those wishing to participate will be contacted by a researcher. Bilingual health advocates will be used where necessary.

- Observations of SP community-based activities. The main researcher will observe a sample of SP community-based activities, paying special attention to the interplay between the individual experience ('emic') and the sociocultural context ('etic') where these activities unfold(58). Observational data on participants' behaviours, characteristics of activities, segments of dialogue between actors and contextual features will be kept in the form of field notes for ulterior analysis.
- Documents. Relevant local SP documentation and policy reports will be accessed for further analysis. We will ask each interviewed policymaker to reference key documents that might be guiding policy in this area.
- Quantitative individual-level data. We will characterise the clinical and socio-demographic features of SP users and the background population eligible for SP (including users of the local 'Healthier You' programme), using data from high quality primary care electronic health records (available from the Clinical Effectiveness Unit, QMUL) and local authority registers.
- Quantitative service-level data. We will gather SP referrers' data by accessing primary care case management systems (EMIS) and/or referral forms. Quantifiable data of local public, private and non-profit institutions and citizens' associations will also be accessed using link worker's referral forms and the records of the organisations under study. We will collect SP costs data to test feasibility of a future scaled up evaluation of SP and T2D prevention frameworks and their potential impact on health systems.

For peer review only

**Table 1. Overview of data source and collection in Stage 2**

| Data source | Data collection |
|---|---|
| **SP service users** | Interviews:<br>• 2 focus groups and 5 in-depth interviews<br>• Purposive sampling, based on socio-economic status, ethnicity, gender, age, participation in SP |
| | Clinical data on all Tower Hamlets residents eligible for SP:<br>• Clinical measurements (BMI, HbA1c)<br>• Diagnosis codes (listed in QOF)<br>• Disease risk (QRISK2, QDScore, prediabetes) |
| | Socio-demographic data on all Tower Hamlets residents eligible for SP:<br>• Age and gender<br>• Ethnicity<br>• Ethnic density of local geography (postcode)<br>• Country of birth<br>• First language spoken<br>• Socio-economic status (Index of Multiple Deprivation, by Lower-layer Super Output Area) |
| **Primary care and link workers** | Interviews:<br>• 3 focus groups and 5 in-depth interviews<br>• Purposive sampling based on professional profile, type of contract, demographic characteristics (sex and age) |
| | SP referrers' quantitative data:<br>• Demographic characteristics (sex and age)<br>• Primary care and link workers' professional profile and background<br>• Type of contract |
| **VCS organizations** | Interviews:<br>• 2 focus groups and 5 in-depth interviews<br>• Purposive sampling based on type of activities, community embeddedness |
| | Ethnographic observations of SP activities |
| | Key documentation: protocols, referral templates, advertising, web pages, etc. |
| | SP providers' quantitative data:<br>• Characteristics of the VCS organisations: available resources, areas of interest<br>• Patients' attendance frequency |
| **SP service** | Overview of the SP service:<br>• Existing referral criteria and reasons for referral<br>• Number of referrals per network, surgery, health worker and profession. |
| | Health economic data, including:<br>• Staff contact<br>• Attendance rates<br>• Facilities use |
| **Policymakers and public health stakeholders** | Interviews:<br>• 5 semi-structured interviews<br>• Snowball sampling |
| | Key documents and policy reports on SP |

For peer review only

(C) Data analysis

Qualitative data will be analysed thematically to identify the patterns and theoretical explanations derived from its concepts, categories and interrelations(59). Data will be analysed according to particular features, but without losing sight of the 'big picture' and the contexts in which it arises(60). In combination with thematic analysis, we will apply a realist logic of analysis to the data to complement, confirm, confront and/or refine the programme theory deriving from the review. Data analysis and interpretation will be reflexively monitored(61). We will seek negative cases and triangulate within the research group for incorporating and maintaining validity, and hence ensuring rigour in the research process(62,63).

Quantitative data will be used to characterise the potential impact of SP on a population at high risk of T2D, and in the context of other existing T2D prevention strategies. These data will not provide a definitive evaluation of SP, but will provide important context for the qualitative research findings and will indicate areas of focus and choice of endpoints (and their feasibility) for future at-scale evaluation. Data will be analysed using descriptive statistics to characterise the clinical and socio-demographic features of patients accessing SP. Comparative statistical tests will be used to look for differences in the SP recipient population compared to the total background population age 18+ (the eligibility criteria for SP is age 18+ and a subjective assessment of need), and to individuals participating in the NHS Type 2 Diabetes prevention programme, Healthier You. Multi-variate analysis will enable to identify potential factors that might be associated with attendance, characterise the uptake and equity of access to SP across the borough, and inform the choice of quantitative outcome measures for a future at-scale evaluation. Considering that 2% of the population receives SP and 1 in 11 people in Tower Hamlets are at high risk of developing T2D (QDScore>20), we anticipate having sufficient power (0.8) to detect a 0.7% absolute difference (35% relative difference) in the likelihood of receiving SP amongst diabetes risk groups, using a type 1 error rate of 0.05.

(D) Synthesis and integration of data

Qualitative findings, framed within CMOCs, will be synthesised according micro, meso and macro levels (and interconnections between levels) that most accurately represent how and why SP programs relevant to T2D prevention might (or might not) 'work'. Quantitative outcomes will be used to provide deeper context to the multi-level program theory. We will undertake a narrative integration of findings(57) and use visual means(64) where appropriate.

For peer review only

**Stage 3. Development of a transferable framework to guide implementation and evaluation of SP relevant to T2D**

Building on the findings of the critical literature review (Stage 1) and the empirical research (Stage 2), we will synthesise a transferable framework for thinking, understanding and evaluating SP programs relevant to populations at high risk of T2D. In order to enhance generalisability, a near final version of the framework will be shared with stakeholders involved in SP programmes relevant to T2D nationwide and further refined based on their inputs.

Similar approaches, such as the NASSS Framework (for theorizing and evaluating Nonadoption, Abandonment, Scale-up, Spread, and Sustainability of health and care technologies)(65), have previously been successfully developed and used in the field of health services research and complex interventions, and have the potential to translate research findings to clinical care, reducing the 'research-implementation divide' that research on complex interventions frequently encounters.

**Ethics and safety**

The study has been approved by the Office for Research Ethics Committees Northern Ireland (reference 20/LO/0713). Standard rules apply for data security, confidentiality and information governance. We will seek informed consent for ethnographic observations during community-based interventions and interviews. We will protect the confidentiality and privacy of participants in focus groups by requesting acceptance of a code of conduct to ensure that personal and private information is not shared or disseminated outside the group.

**Patient and public involvement**

Patient and Public Involvement (PPI) is central to the development of this project and for the plans on how it will be executed. To date, this involvement has come from several groups, including local east London community members (through pilot work to identify the research questions, scope and acceptability), the QMUL-Barts Health Diabetes Lay Research Panel, as well as using Diabetes UK resources including the recent James Lind Alliance T2D priority setting partnership (66). As previously explained, our 'stakeholders' advisory group' will also help to enhance the practical relevance and impact of our research.

**Dissemination and project outputs**

For peer review only

Dissemination will be undertaken as a continuous process and be facilitated by the stakeholders' group. We anticipate that the transferable framework developed in Stage 3 will constitute a key output. We will develop workshops, presentations and summary documents to make it accessible to potential users. Senior policy contacts, who are represented on our stakeholders' group, will be ideally placed to facilitate its wide-reaching dissemination and implementation. We plan outputs for four main audiences.

- For service providers (including primary care workers, social prescribers and VCS), we will provide guidance on how to evaluate and improve local SP schemes.

- For people living in areas at high risk of T2D and community stakeholders, we will develop lay summaries and user-friendly versions of all our findings.

- For policy makers and strategic decision makers, we will produce succinct and accessible briefing papers of key findings aimed at informing prevailing policy and commissioning decisions.

- For the academic community, we will produce research publications in peer-reviewed, open access journals and conference presentations. The whole proposed research will constitute the core contents of a PhD thesis, whose submission and defence will be accomplished from the QMUL.

**DISCUSSION**

There is an increasing interest in SP as a means of addressing the wider social determinants behind escalating burden of long-term conditions, such as T2D, and health inequalities. As part of the NHS Long Term Plan(1), NHS England plans to recruit 1,000 SP link workers so that the service is available in every GP practice by 2024(67).

Despite widespread policy support and proliferation, evidence for the effectiveness of SP is currently sparse, its implementation is heterogeneous and complex, and it incorporates little robust evaluation across health domains(68). Some of the recent studies on SP have used novel methodological approaches to better understand 'how', 'why' and 'in what circumstances' it might or (might not) work(4,69,70). Qualitative research studies have also provided valuable insight into the processes of program implementation and delivery(71-74). However, no previous studies have addressed and evaluated SP in the context of dynamic, interconnected,

For peer review only

multilevel systems. Nor have they 'critically' analysed this context to purposively unfold the tensions, contradictions and competing interests that can potentially shape service delivery.

This study brings a novel methodological approach to investigate whether SP may contribute to comprehensive, community embedded T2D prevention strategies in primary care and its potential impact on populations at high risk. Our multilevel analytic approach will potentially illuminate the complexity of SP and the system in which it operates (including interpersonal, organisational, policy contexts). It is envisaged that project outcomes will inform the design, implementation and evaluation of SP initiatives relevant to T2D, by understanding when, how and in what circumstances this service model might best be introduced.

**Authors' contributions:** SCL and SF conceptualised the study with input from MC and TG. SCL wrote the first draft of this manuscript with significant input from SF, MC, and TG. All authors have read and approved the final manuscript.

**Funding:** This work is supported by the London Interdisciplinary Social Sciences Doctoral Training Partnership and Currier's Millennium Healthcare Bursary

**Disclaimer:** The views and opinions expressed therein are those of the authors and do not necessarily reflect those of the London Interdisciplinary Social Sciences Doctoral Training Partnership, Currier's Millennium Healthcare Bursary, National Health Service or the Department of Health.

**Competing interests:** None declared

**Patient consent:** The submitted paper is a study protocol.

**Ethics approval:** Office for Research Ethics Committees Northern Ireland (reference 20/LO/0713).

**Data sharing statement:** Given this is a study protocol, no data are available to be shared yet.

For peer review only

**REFERENCES:**

1. National Health Service (NHS). NHS Long Term Plan. London, UK; 2019. Available from: https://www.longtermplan.nhs.uk
2. Drinkwater C, Wildman J, Moffatt S. Social prescribing. BMJ. 2019 Mar 28;364:l1285.
3. Moffatt S, Steer M, Lawson S, et al. Link Worker social prescribing to improve health and well-being for people with long-term conditions: qualitative study of service user perceptions. BMJ Open. 2017;7(7):e015203–e015203.
4. Bertotti M, Frostick C, Hutt P, et al. A realist evaluation of social prescribing: an exploration into the context and mechanisms underpinning a pathway linking primary care with the voluntary sector. Prim Heal Care Res Dev. 2018;19(3):232–45.
5. University of York. Centre for Reviews and Dissemination. Evidence to inform the commissioning of social prescribing. 2015. Available from: https://www.york.ac.uk/media/crd/Ev%20briefing_social_prescribing.pdf
6. Social Prescribing Network. Report of the annual social prescribing network conference. London: University of Westminster; 2016.
7. Woodall J, Trigwell J, Bunyan A-M, et al. Understanding the effectiveness and mechanisms of a social prescribing service: a mixed method analysis. BMC Heal Serv Res. 2018;18(1):604.
8. Public Health England. Effectiveness of social prescribing - An evidence synthesis. London: PHE publications; 2019.
9. Bickerdike L, Booth A, Wilson PM, et al. Social prescribing: less rhetoric and more reality. A systematic review of the evidence. BMJ Open. 2017;7(4):e013384.
10. Gottlieb LM, Wing H, Adler NE. A Systematic Review of Interventions on Patients' Social and Economic Needs. Am J Prev Med. 2017 Nov;53(5):719–29.
11. Pescheny J V, Randhawa G, Pappas Y. The impact of social prescribing services on service users: a systematic review of the evidence. Eur j public Heal. 2019.
12. Polley M, Bertotti M, Kimberlee R, et al. A review of the evidence assessing impact of social prescribing on healthcare demand and cost implications. London: University of Westminster; 2017.
13. Pavey TG, Taylor AH, Fox KR, et al. Effect of exercise referral schemes in primary care on physical activity and improving health outcomes: systematic review and meta-analysis. BMJ. 2011 Nov 4;343:d6462.
14. Chatterjee HJ, Camic PM, Lockyer B, et al. Non-clinical community interventions: a

For peer review only

systematised review of social prescribing schemes. Arts Health. 2018 May 4;10(2):97–123.

15. Greenhalgh T, Papoutsi C. Studying complexity in health services research: desperately seeking an overdue paradigm shift. BMC Med. 2018 [cited 2018 Jun 22];16(95).
16. Petticrew M. Public health evaluation: epistemological challenges to evidence production and use. Evid Policy A J Res Debate Pract. 2013 Jan 28;9(1):87–95.
17. Marchal B, Westhorp G, Wong G, et al. Realist RCTs of complex interventions – An oxymoron. Soc Sci Med. 2013 Oct;94:124–8.
18. Cartwright N. Knowing what we are talking about: why evidence doesn't always travel. Evid Policy A J Res Debate Pract. 2013 Jan 28;9(1):97–112.
19. Pilkington K, Loef M, Polley M. Searching for Real-World Effectiveness of Health Care Innovations: Scoping Study of Social Prescribing for Diabetes. J Med Internet Res. 2017;19(2):e20–e20
20. Moffatt S, Wildman J, Pollard TM, et al. Evaluating the impact of a community-based social prescribing intervention on people with type 2 diabetes in North East England: Mixed-methods study protocol. BMJ Open. 2019;9(1).
21. NICE guideline [NG28]. Type 2 diabetes in adults: management. 2019
22. Coen SE. Connecting qualitative research on exercise and environment to public health agendas requires an equity lens. Health Place. 2018 Sep;53:264–7.
23. Darmon N, Drewnowski A. Does social class predict diet quality? Am J Clin Nutr. 2008 May 1;87(5):1107–17.
24. Espelt A, Arriola L, Borrell C, et al. Socioeconomic Position and Type 2 Diabetes Mellitus in Europe 1999- 2009: a Panorama of Inequalities. Curr Diabetes Rev. 2011 May 1;7(3):148–58.
25. Agardh E, Allebeck P, Hallqvist J, et al. Type 2 diabetes incidence and socio-economic position: a systematic review and meta-analysis. Int J Epidemiol. 2011 Jun 1;40(3):804–18.
26. Unwin N, Whiting D, Roglic G. Social determinants of diabetes and challenges of prevention. Lancet. 2010 Jun;375(9733):2204–5.
27. England NHS. NHS Diabetes Prevention Programme (NHS DPP). London;2015. Available from: https://www.england.nhs.uk/ourwork/qual-clin-lead/diabetes-prevention/
28. Barry E, Roberts S, Oke J, et al. Efficacy and effectiveness of screen and treat policies in prevention of type 2 diabetes: systematic review and meta-analysis of

screening tests and interventions. BMJ. 2017 Jan 4;i6538.

29. Davies MJ, Gray LJ, Troughton J, et al. A community based primary prevention programme for type 2 diabetes integrating identification and lifestyle intervention for prevention: the Let's Prevent Diabetes cluster randomised controlled trial. Prev Med (Baltim). 2016 Mar;84:48–56.

30. Valabhji J, Barron E, Bradley D, et al. Early Outcomes From the English National Health Service Diabetes Prevention Programme. Diabetes Care. 2020;43(1):152–60.

31. Barry E, Greenhalgh T, Fahy N. How are health-related behaviours influenced by a diagnosis of pre-diabetes? A meta-narrative review. BMC Med. 2018 Dec 27;16(1):121.

32. Barry E, Roberts S, Finer S, et al. Time to question the NHS diabetes prevention programme. BMJ. 2015 Sep 7;h4717.

33. Tower Hamlets Joint Strategic Needs Assessment Reference Group. Joint Strategic Needs Assessment. Summary Document. Life, Health and Wellbeing in Tower Hamlets. London, UK; 2016. Available from: https://www.towerhamlets.gov.uk/Documents/Public-Health/JSNA/JSNA_Summary.pdf

34. Tower Hamlets Joint Strategic Needs Assessment Reference Group. Joint Strategic Needs Assessment. Type 2 Diabetes 'Factsheet'. London, UK; 2015. Available from: https://www.towerhamlets.gov.uk/Documents/Public-Health/JSNA/Type_2_diabetes.pdf

35. Mole G. Health Equity in Primary Care in East London and the City: Data analysis to inform Joint Strategic Needs Assessment. London, UK; 2014. Available from: www.towerhamlets.gov.uk/Documents/Public-Health/TH-JSNA-Health-Equity-in-Primary-Care-2014.pdf

36. Noble D, Smith D, Mathur R, et al. Feasibility study of geospatial mapping of chronic disease risk to inform public health commissioning. BMJ Open. 2012 Feb 15;2(1):e000711.

37. Mathur R, Noble D, Smith D, et al. Quantifying the risk of type 2 diabetes in East London using the QDScore: a cross-sectional analysis. Br J Gen Pract. 2012 Oct 1;62(603):e663–70.

38. Dreyer G, Hull S, Mathur R, et al. Progression of chronic kidney disease in a multi-ethnic community cohort of patients with diabetes mellitus. Diabet Med. 2013 Aug;30(8):956–63.

39. London Borough of Tower Hamlets. Voluntary and Community Sector Strategy

For peer review only

2016-2019. London, UK; 2016. Available from: https://www.towerhamlets.gov.uk/Documents/Strategy-and-performance/Draft_Voluntary_and_Community_Sector_Strategy_2016_2019.pdf

40. Ferguson K, Hogarth S. Social prescribing in Tower Hamlets: evaluation of borough-wide roll-out. London, UK; 2018. Available from: https://www.towerhamletstogether.com/resource-library

41. Sayer A. Realism and social science. London: Sage Publications; 2000.

42. Bhaskar R. A realist theory of science. 2nd ed. Brighton: Harvester Press; 1978.

43. Krieger N. Epidemiology and the web of causation: Has anyone seen the spider? Soc Sci Med. 1994 Oct;39(7):887–903.

44. Pawson R, Tiley N. Realistic evaluation. London: Sage; 1997.

45. Pawson R. The Science of Evaluation: A Realist Manifesto. London: Sage; 2013. 22–37 p.

46. Merton R. On Theoretical Sociology. Five Essays, Old and New. New York: The Free Press; 1967.

47. Jagosh J, Bush PL, Salsberg J, et al. A realist evaluation of community-based participatory research: partnership synergy, trust building and related ripple effects. BMC Public Health. 2015 Dec 30;15(1):725.

48. Wong G, Greenhalgh T, Westhorp G, et al. RAMESES publication standards: realist syntheses. BMC Med. 2013;11(21).

49. Wong G, Westhorp G, Manzano A, et al. RAMESES II reporting standards for realist evaluations. BMC Med. 2016;14(1):96.

50. Wong G, Greenhalgh T, Westhorp G, et al. Development of methodological guidance, publication standards and training materials for realist and meta-narrative reviews: the RAMESES (Realist And Meta-narrative Evidence Syntheses – Evolving Standards) project. Heal Serv Deliv Res. 2014;2:1–252.

51. Pawson R, Greenhalgh T, Harvey G WK. Realist Synthesis : An Introduction Realist. ESRC research methods programme Manchester: University of Manchester. 2014.

52. Greenhalgh T, Peacock R. Effectiveness and efficiency of search methods in systematic reviews of complex evidence: audit of primary sources. BMJ. 2005 Nov 5;331(7524):1064–5.

53. Maxwell J, Mittapalli K. Realism as a Stance for Mixed Methods Research. In: Tashakkori A, Teddlie C, editors. Handbook of Mixed Methods in Social and Behavioral Research. 2nd edition. Thousand Oaks, CA: Sage; 2010. p. 145–67.

54. Headley MG, Plano Clark VL. Multilevel Mixed Methods Research Designs:

For peer review only

Advancing a Refined Definition. J Mix Methods Res. 2019 May 4;155868981984441.

55. Tariq S, Woodman J. Using mixed methods in health research. JRSM Short Rep. 2013;4(6): 2042533313479197.
56. Creswell JW, Plano Clark VL. Designing and Conducting Mixed Methods Research. Thousand Oaks, CA: Sage; 2007.
57. Fetters MD, Curry LA, Creswell JW. Achieving Integration in Mixed Methods Designs-Principles and Practices. Health Serv Res. 2013 Dec;48(6pt2):2134–56.
58. Hammersley M, Atkinson P. Ethnography: Principles in Practice. Third ed. London: Routledge; 2007.
59. Green J, Thorogood N. Qualitative methods for health research. 2nd ed. London: Sage; 2009.
60. Silverman D. Interpreting Qualitative Data. A Guide to the Principles of Qualitative Research. 4th ed. London: Sage; 2011.
61. Finlay L. ‘Rigour’, ‘Ethical Integrity’ or ‘Artistry’? Reflexively Reviewing Criteria for Evaluating Qualitative Research. Br J Occup Ther. 2006 Jul 5;69(7):319–26.
62. Calderón C. Assessing the Quality of Qualitative Health Research: Criteria, Process and Writing. Forum Qual Soc Res. 2009;10(2:Art 17.).
63. Crabtree B, Miller W. Doing Qualitative Research. Thousand Oaks, CA: Sage; 2000.
64. Plano Clark V, Sanders K. The use of visual displays in mixed methods research: Strategies for effectively integrating the quantitative and qualitative components of a study. In: McCrudden M, Schaw G, Buckendahl C, editors. Use of visual displays in research and testing: Coding, interpreting, and reporting data. Charlotte, NC: Information Age Publishing; 2015. p. 177–206.
65. Greenhalgh T, Wherton J, Papoutsi C, et al. Beyond Adoption: A New Framework for Theorizing and Evaluating Nonadoption, Abandonment, and Challenges to the Scale-Up, Spread, and Sustainability of Health and Care Technologies. J Med Internet Res. 2017 Nov 1;19(11):e367.
66. Finer S, Robb P, Cowan K, et al. Setting the top 10 research priorities to improve the health of people with Type 2 diabetes: a Diabetes UK-James Lind Alliance Priority Setting Partnership. Diabet Med. 2018;35(7):862–70.
67. Hancock M. Keynote Address. Paper presented at the Social prescribing: Coming of age. London: King's Fund. 2018. Available from: https://www.kingsfund.org.uk/events/social-prescribing
68. Husk K, Elston J, Gradinger F, et al. Social prescribing: where is the evidence? Br J Gen Pr. 2019;69(678):6–7.

For peer review only

69. Husk K, Blockley K, Lovell R, et al. What approaches to social prescribing work, for whom, and in what circumstances? A realist review. Heal Soc Care Community. 2019.

70. Tierney S, Wong G, Roberts N, et al. Supporting social prescribing in primary care by linking people to local assets: a realist review. BMC Med. 2020 Dec 13;18(1):49.

71. South J, Higgins TJ, Woodall J, et al. Can social prescribing provide the missing link? Prim Heal Care Res Dev. 2008 Oct 4;9(4):310–8.

72. White JM, Cornish F, Kerr S. Front-line perspectives on 'joined-up' working relationships: a qualitative study of social prescribing in the west of Scotland. Heal Soc Care Community. 2017;25(1):194-203.

73. Skivington K, Smith M, Chng NR, et al. Delivering a primary care-based social prescribing initiative: a qualitative study of the benefits and challenges. Br J Gen Pr. 2018;68(672):e487–94.

74. Wildman JM, Moffatt S, Steer M, et al. Service-users' perspectives of link worker social prescribing: a qualitative follow-up study. BMC Public Health. 2019;19(1):98.

For peer review only

**FIGURE LEGEND**

**Figure 1.** Multi-level and dynamic realist framework

**Figure 2.** Overview of the study design

For peer review only

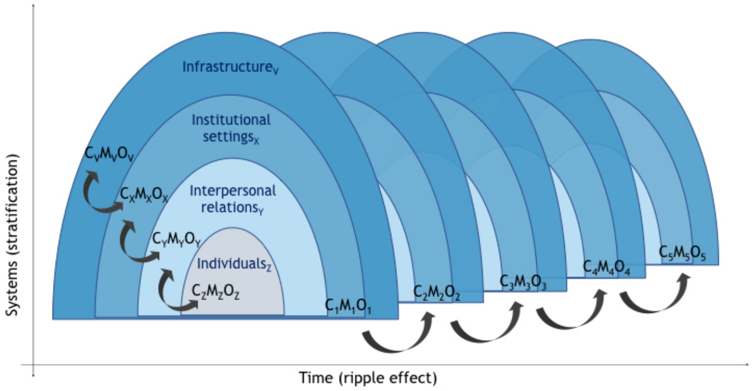


Figure 1. Multi-level realist framework

338x190mm (72 x 72 DPI)

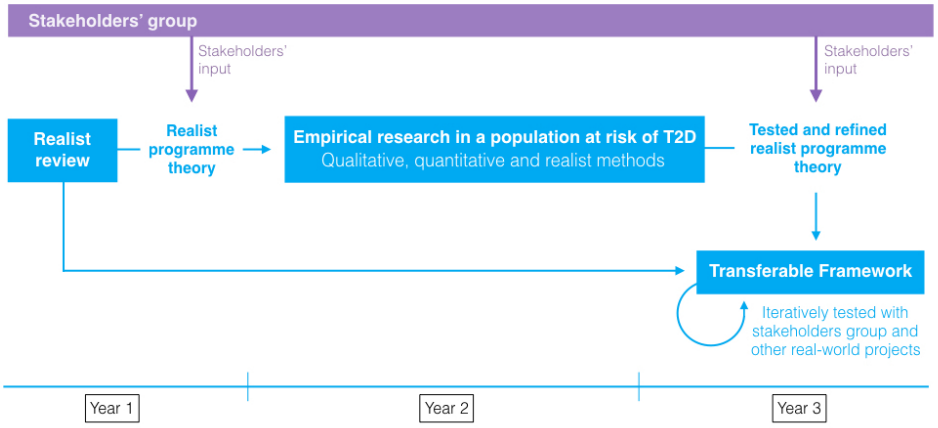


Figure 2. Overview of the study design

361x270mm (72 x 72 DPI)

Appendix 1. Online electronic databases:

- MEDLINE (Ovid)
- EMBASE (Ovid)
- PsycINFO (Ovid)
- Scopus (Elsevier)
- Web of Science (Clarivate Analytics)
- CINAHL Plus (EBSCO)
- PubMed (NCBI)
- International Bibliography of the Social Sciences - IBSS (ProQuest)
- The Cochrane Database of Systematic Reviews (The Cochrane Library)
- Campbell Collaboration
- Open Grey (INISR-CNRS)
- LILACS (BIREME)
- King's Fund Database

For peer review only

BMJ Open

# Could social prescribing contribute to type 2 diabetes prevention in populations at high risk? Protocol for a realist multi-level, mixed-methods review and evaluation.

| | |
|---|---|
| Journal: | *BMJ Open* |
| Manuscript ID | bmjopen-2020-042303.R1 |
| Article Type: | Protocol |
| Date Submitted by the Author: | 08-Nov-2020 |
| Complete List of Authors: | Calderón Larrañaga, Sara; Queen Mary University of London,<br>Clinch , Megan; Barts and the London School of Medicine and Dentistry, Institute of Population Health Sciences<br>Greenhalgh, Trisha; University of Oxford, Nuffield Department of Primary Care Health Sciences<br>Finer, Sarah; Barts and the London School of Medicine and Dentistry, Queen Mary University of London, Blizard Institute |
| <b>Primary Subject Heading</b>: | General practice / Family practice |
| Secondary Subject Heading: | Health services research, Qualitative research, Public health, Diabetes and endocrinology |
| Keywords: | PRIMARY CARE, SOCIAL MEDICINE, DIABETES & ENDOCRINOLOGY |
| | |

SCHOLARONE™
Manuscripts

*I, the Submitting Author has the right to grant and does grant on behalf of all authors of the Work (as defined in the below author licence), an exclusive licence and/or a non-exclusive licence for contributions from authors who are: i) UK Crown employees; ii) where BMJ has agreed a CC-BY licence shall apply, and/or iii) in accordance with the terms applicable for US Federal Government officers or employees acting as part of their official duties; on a worldwide, perpetual, irrevocable, royalty-free basis to BMJ Publishing Group Ltd ("BMJ") its licensees and where the relevant Journal is co-owned by BMJ to the co-owners of the Journal, to publish the Work in this journal and any other BMJ products and to exploit all rights, as set out in our licence.*

*The Submitting Author accepts and understands that any supply made under these terms is made by BMJ to the Submitting Author unless you are acting as an employee on behalf of your employer or a postgraduate student of an affiliated institution which is paying any applicable article publishing charge ("APC") for Open Access articles. Where the Submitting Author wishes to make the Work available on an Open Access basis (and intends to pay the relevant APC), the terms of reuse of such Open Access shall be governed by a Creative Commons licence – details of these licences and which Creative Commons licence will apply to this Work are set out in our licence referred to above.*

*Other than as permitted in any relevant BMJ Author's Self Archiving Policies, I confirm this Work has not been accepted for publication elsewhere, is not being considered for publication elsewhere and does not duplicate material already published. I confirm all authors consent to publication of this Work and authorise the granting of this licence.*

For peer review only

**TITLE:**

Could social prescribing contribute to type 2 diabetes prevention in populations at high risk? Protocol for a realist multi-level, mixed-methods review and evaluation.

**CORRESPONDING AUTHOR:** Sara Calderón-Larrañaga, s.calderon@qmul.ac.uk. Postal address: 58 Turner St, Whitechapel, London E1 2AB.

**AUTHORS**: Sara Calderón-Larrañaga (1,2), Megan Clinch (1), Trish Greenhalgh (3), Sarah Finer (1,4).

1. Centre for Primary Care and Mental Health, Institute of Population Health Sciences, Queen Mary University of London. London, UK
2. Bromley By Bow Health Partnership. London, UK
3. Nuffield Department of Primary Care Health Sciences, University of Oxford, Oxford, UK
4. Barts Health NHS Trust, London, UK

**WORD COUNT:** 3,611

**KEYWORDS:** social prescribing, type 2 diabetes, primary health care, realist methodology

For peer review only

**ABSTRACT:**

**Introduction.** Social Prescribing is an innovation being widely adopted within the UK National Health Service Policy as a way of addressing the social determinants of long-term conditions, such as type 2 diabetes (T2D). It generally involves linking patients in primary care with non-medical community-based interventions. Despite widespread national support, evidence for the effectiveness of social prescribing is both insufficient and contested. In this study, we will investigate whether social prescribing can contribute to T2D prevention and, if so, when, how and in what circumstances it might best be introduced.

**Methods and analysis.** We will draw on realist evaluation to investigate the complex interpersonal, organizational, social and policy context in which social prescribing relevant to T2D prevention is implemented. We will set up a stakeholders' group to advise us throughout the study, which will be conducted over 3 interconnected stages. In Stage 1, we will undertake a realist review to synthesize the current evidence base for social prescribing. In Stage 2, we will investigate how social prescribing relevant to people at high risk of T2D 'works' in a multi-ethnic, socio-economically diverse community and any interactions with existing T2D prevention services using qualitative, quantitative and realist methods. In Stage 3 and building on previous stages, we will synthesise a 'transferable framework' that will guide implementation and evaluation of social prescribing relevant to T2D prevention at scale.

**Ethics and Dissemination.** National Health Service ethics approval has been granted (reference 20/LO/0713). This project will potentially inform the adaptation of social prescribing services to better meet the needs of people at high risk of T2D in socioeconomically deprived areas. Findings may also be transferable to other long-term conditions. Dissemination will be undertaken as a continuous process, supported by the stakeholder group. Tailored outputs will target the following audiences: (1) service providers and commissioners; (2) people at high risk of T2D and community stakeholders; and (3) policy and strategic decision makers.

**Registration details.** PROSPERO (CRD42020196259)

For peer review only

**STRENGTHS AND LIMITATIONS OF THIS STUDY:**

- Our study contributes to a much-needed evidence base for both social prescribing (SP) and its real-world implementation in specific populations, such as those at high risk of type 2 diabetes (T2D)

- The realist methodology will enable to evaluate existing SP programmes within the wider relational, organisational and policy context where they are delivered, including any potential interactions with existing NHS T2D prevention services

- We will seek participatory involvement from our stakeholders' advisory group throughout the study, which will strengthen the practical relevance and transferability of findings

- The study does not intend to generate an effect size, but findings may guide the implementation and evaluation of SP initiatives relevant to T2D prevention at scale

For peer review only

**INTRODUCTION:**

Social Prescribing (SP) is an innovation being widely adopted in the UK, including at policy-level within the National Health Service (NHS) 'Long Term Plan'(1). Although its definition varies, SP generally involves linking patients in primary care with community services offering employment, housing or financial advice, as well as a range of 'healthy lifestyle' activities, such as cooking classes, weight management or exercise programmes(2). Health and lifestyle issues are supposed to be addressed alongside socio-economic matters in an integrated way, usually with the help of a 'social prescriber' (also called 'link worker') who acts as link between health professionals and the wide range of community and voluntary services relevant to patients' situation(3,4). Activities are typically non-medicalised, provided locally and potentially more accessible and culturally appropriate to the local communities(5).

Proponents of SP suggest it may improve health outcomes and redress health inequalities by addressing patients' social and behavioural determinants of health(6). It is also argued that SP could improve the efficient use of NHS and social care resources by enhancing self-care and strengthened community networks(7). However, current evidence base for SP is both scarce and contested. A recent systematic review published by Public Health England comprising 8 UK studies (1 cluster RCT and 7 pre/post-test studies) found no clear evidence for effectiveness of SP schemes(8). In an earlier systematic review, Bickerdike and colleagues did not find enough evidence to prove either success or value for money across 15 studies(9). Further systematic reviews on SP have failed to report consistent health, service utilization or cost results(9-14).

Authors suggest that the evidence gap on SP may be, in part, due to inadequate methodological approaches to gathering, assessing and/or synthesising data(2). Because SP interventions have fuzzy boundaries, multiple and inter-related components and are extremely sensitive to the context, conventional research designs (namely randomised controlled trials (RCTs) and/or previous systematic reviews) may not be the most appropriate approach to evaluation (15-17). Although RCTs would enable to compare the effectiveness of different variations of SP programmes, the methodological requirements to do so could impede the analysis of the interplay between programme implementation, the individuals involved, the programme's context and the wider social environment(18). Novel, complexity-informed methodological approaches are therefore needed to understand 'why', 'for whom', and 'in what context' SP programs might (or might not) work(15,19).



For peer review only - http://bmjopen.bmj.com/site/about/guidelines.xhtml

There is also a need to evaluate the role of SP in specific patient groups and populations(20,21). This study will use type 2 diabetes (T2D) prevention as an exemplar and investigate the impact of SP in populations at high risk of the condition based on the following considerations. First, T2D is a major public health concern, associated with reduced quality of life and life expectancy, and considerable socioeconomic consequences(22). Although individual behavioural risk factors, such as physical inactivity and poor nutrition, are important determinants of T2D risk, these are heavily influenced and constrained by higher-level social and demographic factors(23,24), which appear to be major driving forces behind escalating T2D epidemics and health inequalities(25,26). For T2D prevention strategies to be effective at population level and in the most high-risk groups, they most likely will need to address these social determinants in addition to focusing purely on individual-level behavioural interventions(27). Second, current NHS delivered T2D prevention programmes, such us 'Healthier You'(28), have not demonstrated high uptake(29). The Healthier You intervention consists of 18 educational sessions across 9 months offered to people at high risk of T2D, but its effectiveness in preventing the condition remains uncertain(30). Additionally, a social gradient exists in those engaging with, and gaining benefit from the Healthier You programme(31). There is significant concern that low uptake and high attrition may, at least in part, reflect the fact that individualised behavioural interventions do not adequately address critical social determinants of T2D(32, 33). Evaluating the impact of SP on a specific health area, such as T2D, will potentially enable to examine whether and how such interventions may be able to complement existing individualised behavioural approaches.

**Setting and context:**

This study will be based in Tower Hamlets, a multi-ethnic inner-city district in the East End of London, UK, where overall and T2D-related health outcomes have proved to be poorer compared to the national average(34) despite high quality care(35). Tower Hamlets is one of the most deprived boroughs in the UK and is characterised by its great ethnic diversity, with over half of all residents having a minority ethnic background(36).

Local health intelligence data suggests that age-adjusted deaths directly attributable to diabetes are 8.93 per 100,000, compared to 5.06 across London and 5.06 across England(35). Rates of T2D in Tower Hamlets are significantly higher amongst sub-populations of lowest socio-economic status and South-Asian population(34,36,37). For instance, South Asians have a risk of developing T2D that is two-fold greater than the local white population(38) and suffer from faster chronic kidney progression when they have diabetes(39). Tower Hamlets has become one



For peer review only - http://bmjopen.bmj.com/site/about/guidelines.xhtml

of the 27 areas across the country chosen to be part of the first wave rollout of the national NHS Diabetes Prevention Programme, 'Healthier You'.

Tower Hamlets is also at the forefront of another innovative service model, SP, that is being rapidly adopted throughout the NHS. Tower Hamlets has a long-established local voluntary and community sector (VCS)(40) who have a long history of delivering health promotion activities, including those relevant to T2D prevention. SP is being rolled out borough-wide by the Clinical Commissioning Group, aimed at strengthening and expanding the partnership between the health and voluntary sectors to all local Primary Health Care centres, building on the structure and experience developed in the pre-existing VCS and SP schemes(41). All patients registered with a Tower Hamlets GP, aged over 18 and expressing a 'non-clinical' support need are eligible for the local SP programme. Each of the borough's 36 primary care settings is linked to at least one 'link worker', whose role ranges from signposting to more intensive approaches involving patients' needs assessments, ongoing support and recommendations of relevant VCS services. (41).'

The proposed research will examine the existing evidence base for SP, and critically evaluate SP implementation and delivery in Tower Hamlets, using the following specific aims and questions:

**Aims:**

1. To investigate the possible impact of SP on populations at high risk of T2D.
2. To inform the design, implementation and evaluation of SP initiatives relevant to people at high risk of T2D.

**Research questions:**

1. To what extent might SP meet the complex health and social needs of socio-economically diverse populations at high risk of T2D?
2. What are the main conditions and mechanisms ('active ingredients') that might explain the success or failure of SP programmes relevant to people at risk of T2D?
3. How do existing SP programmes address questions of implementation and evaluation, and what are the knowledge gaps critical to understanding their success or failure within a health system?

**METHODS:**



For peer review only - http://bmjopen.bmj.com/site/about/guidelines.xhtml

**Theoretical and conceptual framework:**

We will draw on realism, a theory-driven methodology that seeks to facilitate deep understanding of how complex interventions, such as SP, work and in what circumstances(42, 43). A central aspect of realism is its analysis of causation, which rejects the standard Humean model of regular succession of events. Realism takes the view that 'what causes something to happen has nothing to do with the number of times we observe it happening, instead identifying causal (also called 'generative') mechanisms of action and explaining how they work, whether they have been activated, and if so under what conditions'(42). Our task as researchers will be to untangle the 'web of causation'(44) identified using this realist approach, and to critically illuminate the underlying structures and mechanisms that might shape and generate empirically observed outcomes.

In order to explore these underlying assumptions, realist methodology proposes the identification of context-mechanism-outcome configurations (CMOc)(45). A CMOc is a hypothesis that the program works (or does not work) (O) because of the action of some underlying mechanisms (M), which only comes into operation in particular contexts (C). '*If* the right processes operate in the right conditions *then* the program will prevail'(46). These kind of theoretical explanations, sufficiently abstract to draw transferable lessons but closed enough to the observable data to enable empirical testing and refinement, are referred to as 'programme theories'(47).

CMOc may link to each other over time, with the outcome of one phase becoming the context of the next configuration in the chain of implementation steps (known as 'ripple effect')(48). Yet, CMOc may also organize concentrically(46). In this study, we propose a layered and dynamic realist framework (Figure 1) to investigate the complex interpersonal, organisational, social and policy context in which SP relevant T2D is implemented, and illuminate key domains and tensions at these different and interconnected levels. A theory-testing strategy will be used to unpack these causal mechanisms and interrelated CMOc. Preliminary theoretical explanations will be made explicit and iteratively tested, confronted and refined using relevant and varied evidence from the primary literature (realist review(49)) and/or empirical data (realist evaluation(50)).

**Research Plan:**

The study will be conducted in three interlinked stages over three years as follows:


For peer review only - http://bmjopen.bmj.com/site/about/guidelines.xhtml

- Stage 1 – Development and refinement of a realist programme theory for SP interventions relevant to populations at high risk of T2D using a critical systematic literature review (realist synthesis).

- Stage 2 – Empirical testing and further refinement of a realist programme theory (guided by Stage 1) in a socio-economically deprived, ethnically-diverse community at high risk of T2D using qualitative, quantitative and realist methods (realist evaluation).

- Stage 3 – Synthesis of secondary (Stage 1) and primary (Stage 2) evidence, involving the development of a transferable framework for guiding implementation and evaluation of SP relevant to T2D prevention at scale.

In parallel and as represented in Figure 2, we will set up a stakeholders' group comprising representatives of the VCS, primary care leaders, members of Public Health England and relevant national charitable organisations (such as SP Network, Deep End Group, and/or the National Academy for Social Prescribing). Via individual and group interviews, the study objectives and ongoing research outcomes will be iteratively shared, leading to discussions that will seek to explore and highlight stakeholders' opinions and concerns about SP, T2D prevention and the emerging programme theory.

**Stage 1. Development and refinement of a realist programme theory (Realist Synthesis)**

Following the Realist And Meta-narrative Evidence Syntheses: Evolving Standards (RAMESES)(49,51), we will undertake a realist review in four iterative stages: (A) Searching for evidence, (B) Selecting and appraising literature, (C) Extracting and organizing data and (D) Synthesizing the evidence and drawing conclusions(52).

(A) Searching for evidence

Searches will be organised and performed in two phases. The main search will be aimed at identifying the rationales for why and how SP might (or might not) play a role in T2D prevention and building preliminary programme theories that will be negotiated within the research and stakeholders' groups. We will then undertake targeted searches aimed at refining these candidate programme theories.

We will use free-text and indexing search terms employed in previous systematic reviews(8,9,20). Terms for the second search will depend on the results from the first phase and will be discussed with a specialist librarian and the review team. We will search for qualitative,



For peer review only - http://bmjopen.bmj.com/site/about/guidelines.xhtml

quantitative and mixed-methods studies in relevant electronic databases and grey literature resources (the search strategy and databases for the main search are specified in Appendix 1). We will also undertake forwards and backwards citation chasing on included studies, hand-searching of key journals, and study author contact where appropriate(53). Results will be exported to Rayyan QCRI for de-deduplication. The searching process will be reproduced by a second reviewer for consistency.

(B) Selecting and appraising articles

Studies will be included if they consider adults (18+) in primary care settings and focus on interventions that link patients in primary care with community-based resources. Studies will be excluded if written in languages other than English, Spanish or French. Inclusion and exclusion criteria will continue to be refined throughout the review as theoretical understanding develops, and until theoretical saturation has been reached. Literature will be appraised based on their relevance at the point of inclusion ('does it contribute to theory building and testing?') and rigour ('are the methods used to generate the relevant data credible and trustworthy?')(51). A 10% random subsample of screening decisions will be reviewed by a second reviewer for consistency.

(C) Extracting and organizing data

The main reviewer will read all included articles, extract descriptive study characteristics and carry out manual coding. Full texts and the coding framework resulting from the manual analysis will then be uploaded into NVivo 11 (qualitative data management software). Initial coding will comprise broad conceptual categories that will progressively be refined both inductively and deductively, and reframed applying a realist logic of analysis. This means that we will work iteratively to identify sections of the text related to contexts (C), mechanisms (M) and outcomes (O) and organize data in level-specific CMOc(52). Again, a 10% random subsample of coded articles will be reviewed by a second reviewer for consistency, and disagreements will be solved by discussion.

(D) Synthesizing the evidence and drawing conclusions

We will use evidence synthesis processes proposed by realist methodology (e.g. juxtaposition of data sources, reconciling 'contradictory' or disconfirming data, consolidation of sources of evidence)(46) to articulate clear and contextualised explanations on how and why certain



For peer review only - http://bmjopen.bmj.com/site/about/guidelines.xhtml

patterns related to SP relevant to T2D prevention occur. These theory-informed, evidence-based, multilevel propositions will guide the empirical realist evaluation and the development of a transferable framework in stages 2 and 3, respectively.

**Stage 2. Empirical testing and further refinement of a realist programme theory (Realist Evaluation)**

(A) Study Design

We will undertake a realist, multi-level and mixed-methods evaluation of an existing SP programme, based in Tower Hamlets, east London (the setting has been described above). The appropriateness and relevance of these approaches in the study and evaluation of complex, multi-dimensional, dynamic phenomena (such as SP) have been well stablished in the literature(54-56).

At microlevel, we will characterise SP users and investigate their experience throughout the SP pathway (from initial referral to subsequent assessment by the link worker and unfolding community-based activities), paying special attention to the dynamic interaction between the different actors involved (patients, primary care clinicians, link workers, members of local community organisations, local authority/public health services). At mesolevel, we will explore how a primary care service and the voluntary and community sector serving a community at high risk of T2D interact and operate to enable SP. We will also study the potential interaction of SP schemes with existing NHS T2D prevention programmes, such us 'Healthier You'. At macrolevel, we will investigate the barriers, facilitators and incentives to supporting SP relevant to T2D within the managerial and political sphere, and explore the interests and significances of SP for policymakers and other key stakeholders. Following a convergent mixed-methods study design, qualitative and quantitative data will be collected and analysed 'in parallel' (during a similar timeframe) using an interactive approach, where data collection and analysis might drive changes in ongoing data collection procedures(57,58).

(B) Data collection

Data will be generated through a number of qualitative and quantitative methods (Table 1):

- Individual and group interviews. We will purposively select SP users living in an area at high risk of T2D (including those who did not turn up for appointments or declined to

For peer review only

engage with the activities), members of VCS organisations involved in SP, as well as local primary care clinicians, link workers and policy makers. Sampling will be guided by the strategies specified in table 1 and the findings of the realist review, as to the characteristics and circumstances that play a role in the success or failure of SP. Sample size will be determined by data saturation. An initial invitation letter and consent form will be provided and those wishing to participate will be contacted by a researcher. Interviews will be arranged at a time and location convenient to the participants, and bilingual health advocates will be used where necessary.

- Observations of SP community-based activities. The main researcher will observe a sample of SP community-based activities, paying special attention to the interplay between the individual experience ('emic') and the sociocultural context ('etic') where these activities unfold(59). Observational data on participants' behaviours, characteristics of activities, segments of dialogue between actors and contextual features will be kept in the form of field notes for ulterior analysis.
- Documents. Relevant local SP documentation and policy reports will be accessed for further analysis. We will ask each interviewed policymaker to reference key documents that might be guiding policy in this area.
- Quantitative individual-level data. We will characterise the clinical and socio-demographic features of SP users and the background population eligible for SP (including users of the local 'Healthier You' programme), using anonymised data from high quality primary care electronic health records (available from the Clinical Effectiveness Unit, Queen Mary University of London) and local authority registers.
- Quantitative service-level data. We will gather SP referrers' data by accessing primary care case management systems (EMIS) and/or referral forms. Quantifiable data of local public, private and non-profit institutions and citizens' associations will also be accessed using link worker's referral forms and the records of the organisations under study. We will collect SP costs data to test feasibility of a future scaled up evaluation of SP and T2D prevention frameworks and their potential impact on health systems.


For peer review only - http://bmjopen.bmj.com/site/about/guidelines.xhtml

**Table 1. Overview of data source and collection in Stage 2**

| Data source | Data collection |
|---|---|
| **SP service users** | Interviews:<br>• Approximately 2 focus groups and 8 in-depth interviews<br>• Purposive sampling, based on socio-economic status, ethnicity, gender, age, participation in SP |
| | Clinical data on all Tower Hamlets residents eligible for SP:<br>• Clinical measurements (HbA1c)<br>• Diagnosis codes (listed in QOF, including hypertension, diabetes, obesity)<br>• Disease risk (QRISK2, QDScore, prediabetes) |
| | Socio-demographic data on all Tower Hamlets residents eligible for SP:<br>• Age and gender<br>• Ethnicity<br>• Ethnic density of local geography (postcode)<br>• Country of birth<br>• First language spoken<br>• Socio-economic status (Index of Multiple Deprivation, by Lower-layer Super Output Area) |
| **Primary care professionals, including link workers** | Interviews:<br>• Approximately 1 focus group and 8 in-depth interviews<br>• Purposive sampling based on professional profile, type of contract, demographic characteristics (age, gender, ethnicity) |
| | SP referrers' quantitative data:<br>• Demographic characteristics (age and gender)<br>• Primary care workers' professional profile and background<br>• Type of contract |
| **VCS organizations** | Interviews:<br>• Approximately 1 focus group and 8 in-depth interviews<br>• Purposive sampling based on type of activities, community embeddedness |
| | Observations of SP activities |
| | Key documentation: protocols, referral templates, advertising, web pages, etc. |
| | SP providers' quantitative data:<br>• Characteristics of the VCS organisations: available resources, areas of interest<br>• Patients' attendance frequency |
| **SP service** | Overview of the SP service:<br>• Existing referral criteria and reasons for referral<br>• Number of referrals per network, surgery, health worker and profession. |
| | Health economic data, including:<br>• Staff contact<br>• Attendance rates<br>• Facilities use |
| **Policymakers and public health stakeholders** | Interviews:<br>• Approximately 5 semi-structured interviews<br>• Snowball sampling |
| | Key documents and policy reports on SP |

For peer review only

(C) Data analysis

Qualitative data will be analysed thematically to identify the patterns and theoretical explanations derived from its concepts, categories and interrelations(60). Data will be analysed according to particular features, but without losing sight of the 'big picture' and the contexts in which it arises(61). In combination with thematic analysis, we will apply a realist logic of analysis to the data to complement, confirm, confront and/or refine the programme theory deriving from the review. Data analysis and interpretation will be reflexively monitored(62). We will seek negative cases and triangulate within the research group for incorporating and maintaining validity, and hence ensuring rigour in the research process(63,64).

Quantitative data will be used to characterise the potential impact of SP on a population at high risk of T2D, and in the context of other existing T2D prevention strategies. These data will not provide a definitive evaluation of SP, but will provide important context for the qualitative research findings and will indicate areas of focus and choice of endpoints (and their feasibility) for future at-scale evaluation. Data will be analysed using descriptive statistics to characterise the clinical and socio-demographic features of patients accessing SP. Comparative statistical tests will be used to look for differences in the SP recipient population compared to the total background population age 18+ (the eligibility criteria for SP is age 18+ and a subjective assessment of need), and to individuals participating in the NHS Type 2 Diabetes prevention programme, Healthier You. Multi-variate analysis will enable to identify potential factors that might be associated with the likelihood of being referred into SP and characterise the uptake and equity of access to the programme across the borough. Considering that 2% of the population receives SP and 1 in 11 people in Tower Hamlets are at high risk of developing T2D (QDScore>20), we anticipate having sufficient power (0.8) to detect a 0.7% absolute difference (35% relative difference) in the likelihood of receiving SP amongst diabetes risk groups, using a type 1 error rate of 0.05.

(D) Synthesis and integration of data

Qualitative findings, framed within CMOCs, will be synthesised according micro, meso and macro levels (and interconnections between levels) that most accurately represent how and why SP programs relevant to T2D prevention might (or might not) 'work'. Quantitative outcomes will be used to provide deeper context to the multi-level program theory. We will undertake a narrative integration of findings(58) and use visual means(65) where appropriate.


For peer review only - http://bmjopen.bmj.com/site/about/guidelines.xhtml

## Stage 3. Development of a transferable framework to guide implementation and evaluation of SP relevant to T2D

Building on the findings of the critical systematic literature review (Stage 1) and the empirical research (Stage 2), we will synthesise a transferable framework for thinking, understanding and evaluating SP programs relevant to populations at high risk of T2D. In order to enhance generalisability, a near final version of the framework will be shared with stakeholders involved in SP programmes relevant to T2D nationwide and further refined based on their inputs.

Similar approaches, such as the NASSS Framework (for theorizing and evaluating Nonadoption, Abandonment, Scale-up, Spread, and Sustainability of health and care technologies)(66), have previously been successfully developed and used in the field of health services research and complex interventions, and have the potential to translate research findings to clinical care, reducing the 'research-implementation divide' that research on complex interventions frequently encounters.

## Ethics and dissemination

HRA and Health and Care Research Wales (HCRW) ethics approval has been granted (reference 20/LO/0713). Standard rules apply for data security, confidentiality and information governance. We will seek informed consent for observations during community-based interventions and interviews. We will protect the confidentiality and privacy of participants in focus groups by requesting acceptance of a code of conduct to ensure that personal and private information is not shared outside the group.

Dissemination of study outputs will be undertaken as a continuous process and be facilitated by the stakeholders' group. We anticipate that the transferable framework developed in Stage 3 will constitute a key output. We will develop workshops, presentations and summary documents to make it accessible to potential users. Senior policy contacts, who are represented on our stakeholders' group, will be ideally placed to facilitate its wide-reaching dissemination and implementation. We plan outputs for four main audiences.

- For service providers (including primary care clinicians, link workers and the VCS), we will provide guidance on how to evaluate and improve local SP schemes.

For peer review only

- For people living in areas at high risk of T2D and community stakeholders, we will develop lay summaries and user-friendly versions of all our findings.

- For policy makers and strategic decision makers, we will produce succinct and accessible briefing papers of key findings aimed at informing prevailing policy and commissioning decisions.

- For the academic community, we will produce research publications in peer-reviewed, open access journals and conference presentations. The whole proposed research will constitute the core contents of a PhD thesis, whose submission and defence will be accomplished from the QMUL.

**Patient and public involvement**

Patient and Public Involvement (PPI) is central to the development of this project and for the plans on how it will be executed. To date, this involvement has come from several groups, including local east London community members (through pilot work to identify the research questions, scope and acceptability), the QMUL-Barts Health Diabetes Lay Research Panel, as well as using Diabetes UK resources including the recent James Lind Alliance T2D priority setting partnership (67). As previously explained, our 'stakeholders' advisory group' will also help to enhance the practical relevance and impact of our research.

**DISCUSSION**

There is an increasing interest in SP as a means of addressing the wider social determinants behind escalating burden of long-term conditions, such as T2D, and health inequalities. As part of the NHS Long Term Plan(1), NHS England plans to recruit 1,000 SP link workers so that the service is available in every GP practice by 2024(68).

Despite widespread policy support and proliferation, evidence for the effectiveness of SP is currently sparse, its implementation is heterogeneous and complex, and it incorporates little robust evaluation across health domains(69). Some of the recent studies on SP have used novel methodological approaches to better understand 'how', 'why' and 'in what circumstances' it might or (might not) work(4,70,71). Qualitative research studies have also provided valuable insight into the processes of program implementation and delivery(72-75). However, no previous studies have addressed and evaluated SP in the context of dynamic, interconnected,

For peer review only

multilevel systems. Nor have they ‘critically’ analysed this context to purposively unfold the tensions, contradictions and competing interests that can potentially shape service delivery.

This study brings a novel methodological approach to investigate whether SP may contribute to comprehensive, community embedded T2D prevention strategies in primary care and its potential impact on populations at high risk. Our multilevel analytic approach will potentially illuminate the complexity of SP and the system in which it operates (including interpersonal, organisational, policy contexts). It is envisaged that project outcomes will inform the design, implementation and evaluation of SP initiatives relevant to T2D, by understanding when, how and in what circumstances this service model might best be introduced.

**Authors’ contributions:** SCL and SF conceptualised the study with input from MC and TG. SCL wrote the first draft of this manuscript with significant input from SF, MC, and TG. All authors have read and approved the final manuscript.

**Funding:** This work is supported by the Economic Social Research Council (ESRC) via the London Interdisciplinary Social Sciences Doctoral Training Partnership (LISS DTP) (reference ES/P000703/1) and the Currier’s Company Millennium Trust (Worshipful Company of Currier's Millennium Trust Bursary Recipient 2020).

**Disclaimer:** The views and opinions expressed therein are those of the authors and do not necessarily reflect those of the London Interdisciplinary Social Sciences Doctoral Training Partnership, Currier’s Millennium Healthcare Bursary, National Health Service or the Department of Health.

**Competing interests:** None declared

**Patient consent:** The submitted paper is a study protocol.

**Ethics approval:** Health Research Authority (HRA) and Health and Care Research Wales (HCRW) ethics approval (reference 20/LO/0713).

**Data sharing statement:** Given this is a study protocol, no data are available to be shared yet.

For peer review only

For peer review only



For peer review only - http://bmjopen.bmj.com/site/about/guidelines.xhtml

**REFERENCES:**


1. National Health Service (NHS). NHS Long Term Plan. London, UK; 2019. Available from: https://www.longtermplan.nhs.uk
2. Drinkwater C, Wildman J, Moffatt S. Social prescribing. BMJ. 2019 Mar 28;364:l1285.
3. Moffatt S, Steer M, Lawson S, et al. Link Worker social prescribing to improve health and well-being for people with long-term conditions: qualitative study of service user perceptions. BMJ Open. 2017;7(7):e015203–e015203.
4. Bertotti M, Frostick C, Hutt P, et al. A realist evaluation of social prescribing: an exploration into the context and mechanisms underpinning a pathway linking primary care with the voluntary sector. Prim Heal Care Res Dev. 2018;19(3):232–45.
5. University of York. Centre for Reviews and Dissemination. Evidence to inform the commissioning of social prescribing. 2015. Available from: https://www.york.ac.uk/media/crd/Ev%20briefing_social_prescribing.pdf
6. Social Prescribing Network. Report of the annual social prescribing network conference. London: University of Westminster; 2016.
7. Woodall J, Trigwell J, Bunyan A-M, et al. Understanding the effectiveness and mechanisms of a social prescribing service: a mixed method analysis. BMC Heal Serv Res. 2018;18(1):604.
8. Public Health England. Effectiveness of social prescribing - An evidence synthesis. London: PHE publications; 2019.
9. Bickerdike L, Booth A, Wilson PM, et al. Social prescribing: less rhetoric and more reality. A systematic review of the evidence. BMJ Open. 2017;7(4):e013384.
10. Gottlieb LM, Wing H, Adler NE. A Systematic Review of Interventions on Patients' Social and Economic Needs. Am J Prev Med. 2017 Nov;53(5):719–29.
11. Pescheny J V, Randhawa G, Pappas Y. The impact of social prescribing services on service users: a systematic review of the evidence. Eur j public Heal. 2019.
12. Polley M, Bertotti M, Kimberlee R, et al. A review of the evidence assessing impact of social prescribing on healthcare demand and cost implications. London: University of Westminster; 2017.
13. Pavey TG, Taylor AH, Fox KR, et al. Effect of exercise referral schemes in primary care on physical activity and improving health outcomes: systematic review and meta-analysis. BMJ. 2011 Nov 4;343:d6462.
14. Chatterjee HJ, Camic PM, Lockyer B, et al. Non-clinical community interventions: a


For peer review only

systematised review of social prescribing schemes. Arts Health. 2018 May 4;10(2):97–123.

15. Greenhalgh T, Papoutsi C. Studying complexity in health services research: desperately seeking an overdue paradigm shift. BMC Med. 2018 [cited 2018 Jun 22];16(95).

16. Petticrew M. Public health evaluation: epistemological challenges to evidence production and use. Evid Policy A J Res Debate Pract. 2013 Jan 28;9(1):87–95.

17. Craig P, Dieppe P, Macintyre S, Michie S, Nazareth I, Petticrew M. Developing and evaluating complex interventions: the new Medical Research Council guidance. BMJ. 2008 Sep 29;a1655.

18. Marchal B, Westhorp G, Wong G, et al. Realist RCTs of complex interventions – An oxymoron. Soc Sci Med. 2013 Oct;94:124–8.

19. Cartwright N. Knowing what we are talking about: why evidence doesn't always travel. Evid Policy A J Res Debate Pract. 2013 Jan 28;9(1):97–112.

20. Pilkington K, Loef M, Polley M. Searching for Real-World Effectiveness of Health Care Innovations: Scoping Study of Social Prescribing for Diabetes. J Med Internet Res. 2017;19(2):e20–e20

21. Moffatt S, Wildman J, Pollard TM, et al. Evaluating the impact of a community-based social prescribing intervention on people with type 2 diabetes in North East England: Mixed-methods study protocol. BMJ Open. 2019;9(1).

22. NICE guideline [NG28]. Type 2 diabetes in adults: management. 2019

23. Coen SE. Connecting qualitative research on exercise and environment to public health agendas requires an equity lens. Health Place. 2018 Sep;53:264–7.

24. Darmon N, Drewnowski A. Does social class predict diet quality? Am J Clin Nutr. 2008 May 1;87(5):1107–17.

25. Espelt A, Arriola L, Borrell C, et al. Socioeconomic Position and Type 2 Diabetes Mellitus in Europe 1999- 2009: a Panorama of Inequalities. Curr Diabetes Rev. 2011 May 1;7(3):148–58.

26. Agardh E, Allebeck P, Hallqvist J, et al. Type 2 diabetes incidence and socio-economic position: a systematic review and meta-analysis. Int J Epidemiol. 2011 Jun 1;40(3):804–18.

27. Unwin N, Whiting D, Roglic G. Social determinants of diabetes and challenges of prevention. Lancet. 2010 Jun;375(9733):2204–5.

28. England NHS. NHS Diabetes Prevention Programme (NHS DPP). London;2015. Available from: https://www.england.nhs.uk/ourwork/qual-clin-lead/diabetes-

For peer review only

prevention/

29. Barry E, Roberts S, Oke J, et al. Efficacy and effectiveness of screen and treat policies in prevention of type 2 diabetes: systematic review and meta-analysis of screening tests and interventions. BMJ. 2017 Jan 4;i6538.
30. Davies MJ, Gray LJ, Troughton J, et al. A community based primary prevention programme for type 2 diabetes integrating identification and lifestyle intervention for prevention: the Let's Prevent Diabetes cluster randomised controlled trial. Prev Med (Baltim). 2016 Mar;84:48–56.
31. Valabhji J, Barron E, Bradley D, et al. Early Outcomes From the English National Health Service Diabetes Prevention Programme. Diabetes Care. 2020;43(1):152–60.
32. Barry E, Greenhalgh T, Fahy N. How are health-related behaviours influenced by a diagnosis of pre-diabetes? A meta-narrative review. BMC Med. 2018 Dec 27;16(1):121.
33. Barry E, Roberts S, Finer S, et al. Time to question the NHS diabetes prevention programme. BMJ. 2015 Sep 7;h4717.
34. Tower Hamlets Joint Strategic Needs Assessment Reference Group. Joint Strategic Needs Assessment. Summary Document. Life, Health and Wellbeing in Tower Hamlets. London, UK; 2016. Available from: https://www.towerhamlets.gov.uk/Documents/Public-Health/JSNA/JSNA_Summary.pdf
35. Tower Hamlets Joint Strategic Needs Assessment Reference Group. Joint Strategic Needs Assessment. Type 2 Diabetes 'Factsheet'. London, UK; 2015. Available from: https://www.towerhamlets.gov.uk/Documents/Public-Health/JSNA/Type_2_diabetes.pdf
36. Mole G. Health Equity in Primary Care in East London and the City: Data analysis to inform Joint Strategic Needs Assessment. London, UK; 2014. Available from: www.towerhamlets.gov.uk/Documents/Public-Health/TH-JSNA-Health-Equity-in-Primary-Care-2014.pdf
37. Noble D, Smith D, Mathur R, et al. Feasibility study of geospatial mapping of chronic disease risk to inform public health commissioning. BMJ Open. 2012 Feb 15;2(1):e000711.
38. Mathur R, Noble D, Smith D, et al. Quantifying the risk of type 2 diabetes in East London using the QDScore: a cross-sectional analysis. Br J Gen Pract. 2012 Oct 1;62(603):e663–70.
39. Dreyer G, Hull S, Mathur R, et al. Progression of chronic kidney disease in a multi-

For peer review only

ethnic community cohort of patients with diabetes mellitus. Diabet Med. 2013 Aug;30(8):956–63.

40. London Borough of Tower Hamlets. Voluntary and Community Sector Strategy 2016-2019. London, UK; 2016. Available from: https://www.towerhamlets.gov.uk/Documents/Strategy-and-performance/Draft_Voluntary_and_Community_Sector_Strategy_2016_2019.pdf

41. Ferguson K, Hogarth S. Social prescribing in Tower Hamlets: evaluation of borough-wide roll-out. London, UK; 2018. Available from: https://www.towerhamletstogether.com/resource-library

42. Sayer A. Realism and social science. London: Sage Publications; 2000.

43. Bhaskar R. A realist theory of science. 2nd ed. Brighton: Harvester Press; 1978.

44. Krieger N. Epidemiology and the web of causation: Has anyone seen the spider? Soc Sci Med. 1994 Oct;39(7):887–903.

45. Pawson R, Tiley N. Realistic evaluation. London: Sage; 1997.

46. Pawson R. The Science of Evaluation: A Realist Manifesto. London: Sage; 2013. 22–37 p.

47. Merton R. On Theoretical Sociology. Five Essays, Old and New. New York: The Free Press; 1967.

48. Jagosh J, Bush PL, Salsberg J, et al. A realist evaluation of community-based participatory research: partnership synergy, trust building and related ripple effects. BMC Public Health. 2015 Dec 30;15(1):725.

49. Wong G, Greenhalgh T, Westhorp G, et al. RAMESES publication standards: realist syntheses. BMC Med. 2013;11(21).

50. Wong G, Westhorp G, Manzano A, et al. RAMESES II reporting standards for realist evaluations. BMC Med. 2016;14(1):96.

51. Wong G, Greenhalgh T, Westhorp G, et al. Development of methodological guidance, publication standards and training materials for realist and meta-narrative reviews: the RAMESES (Realist And Meta-narrative Evidence Syntheses – Evolving Standards) project. Heal Serv Deliv Res. 2014;2:1–252.

52. Pawson R, Greenhalgh T, Harvey G WK. Realist Synthesis : An Introduction Realist. ESRC research methods programme Manchester: University of Manchester. 2014.

53. Greenhalgh T, Peacock R. Effectiveness and efficiency of search methods in systematic reviews of complex evidence: audit of primary sources. BMJ. 2005 Nov 5;331(7524):1064–5.

54. Maxwell J, Mittapalli K. Realism as a Stance for Mixed Methods Research. In:



For peer review only - http://bmjopen.bmj.com/site/about/guidelines.xhtml

Tashakkori A, Teddlie C, editors. Handbook of Mixed Methods in Social and Behavioral Research. 2nd edition. Thousand Oaks, CA: Sage; 2010. p. 145–67.

55. Headley MG, Plano Clark VL. Multilevel Mixed Methods Research Designs: Advancing a Refined Definition. J Mix Methods Res. 2019 May 4;155868981984441.

56. Tariq S, Woodman J. Using mixed methods in health research. JRSM Short Rep. 2013;4(6): 2042533313479197.

57. Creswell JW, Plano Clark VL. Designing and Conducting Mixed Methods Research. Thousand Oaks, CA: Sage; 2007.

58. Fetters MD, Curry LA, Creswell JW. Achieving Integration in Mixed Methods Designs-Principles and Practices. Health Serv Res. 2013 Dec;48(6pt2):2134–56.

59. Hammersley M, Atkinson P. Ethnography: Principles in Practice. Third ed. London: Routledge; 2007.

60. Green J, Thorogood N. Qualitative methods for health research. 2nd ed. London: Sage; 2009.

61. Silverman D. Interpreting Qualitative Data. A Guide to the Principles of Qualitative Research. 4th ed. London: Sage; 2011.

62. Finlay L. 'Rigour', 'Ethical Integrity' or 'Artistry'? Reflexively Reviewing Criteria for Evaluating Qualitative Research. Br J Occup Ther. 2006 Jul 5;69(7):319–26.

63. Calderón C. Assessing the Quality of Qualitative Health Research: Criteria, Process and Writing. Forum Qual Soc Res. 2009;10(2:Art 17.).

64. Crabtree B, Miller W. Doing Qualitative Research. Thousand Oaks, CA: Sage; 2000.

65. Plano Clark V, Sanders K. The use of visual displays in mixed methods research: Strategies for effectively integrating the quantitative and qualitative components of a study. In: McCrudden M, Schaw G, Buckendahl C, editors. Use of visual displays in research and testing: Coding, interpreting, and reporting data. Charlotte, NC: Information Age Publishing; 2015. p. 177–206.

66. Greenhalgh T, Wherton J, Papoutsi C, et al. Beyond Adoption: A New Framework for Theorizing and Evaluating Nonadoption, Abandonment, and Challenges to the Scale-Up, Spread, and Sustainability of Health and Care Technologies. J Med Internet Res. 2017 Nov 1;19(11):e367.

67. Finer S, Robb P, Cowan K, et al. Setting the top 10 research priorities to improve the health of people with Type 2 diabetes: a Diabetes UK-James Lind Alliance Priority Setting Partnership. Diabet Med. 2018;35(7):862–70.

68. Hancock M. Keynote Address. Paper presented at the Social prescribing: Coming of age. London: King's Fund. 2018. Available from:

For peer review only

https://www.kingsfund.org.uk/events/social-prescribing

69. Husk K, Elston J, Gradinger F, et al. Social prescribing: where is the evidence? Br J Gen Pr. 2019;69(678):6–7.

70. Husk K, Blockley K, Lovell R, et al. What approaches to social prescribing work, for whom, and in what circumstances? A realist review. Heal Soc Care Community. 2019.

71. Tierney S, Wong G, Roberts N, et al. Supporting social prescribing in primary care by linking people to local assets: a realist review. BMC Med. 2020 Dec 13;18(1):49.

72. South J, Higgins TJ, Woodall J, et al. Can social prescribing provide the missing link? Prim Heal Care Res Dev. 2008 Oct 4;9(4):310–8.

73. White JM, Cornish F, Kerr S. Front-line perspectives on 'joined-up' working relationships: a qualitative study of social prescribing in the west of Scotland. Heal Soc Care Community. 2017;25(1):194-203.

74. Skivington K, Smith M, Chng NR, et al. Delivering a primary care-based social prescribing initiative: a qualitative study of the benefits and challenges. Br J Gen Pr. 2018;68(672):e487–94.

75. Wildman JM, Moffatt S, Steer M, et al. Service-users' perspectives of link worker social prescribing: a qualitative follow-up study. BMC Public Health. 2019;19(1):98.

For peer review only

**FIGURE LEGEND**

**Figure 1.** Multi-level and dynamic realist framework

**Figure 2.** Overview of the study design

For peer review only

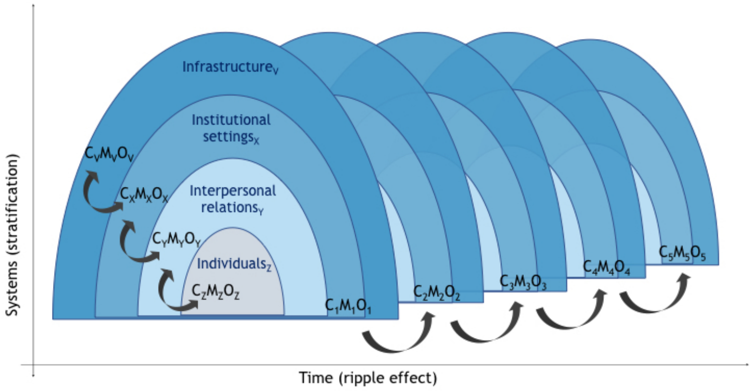


Figure 1. Multi-level realist framework

338x190mm (72 x 72 DPI)

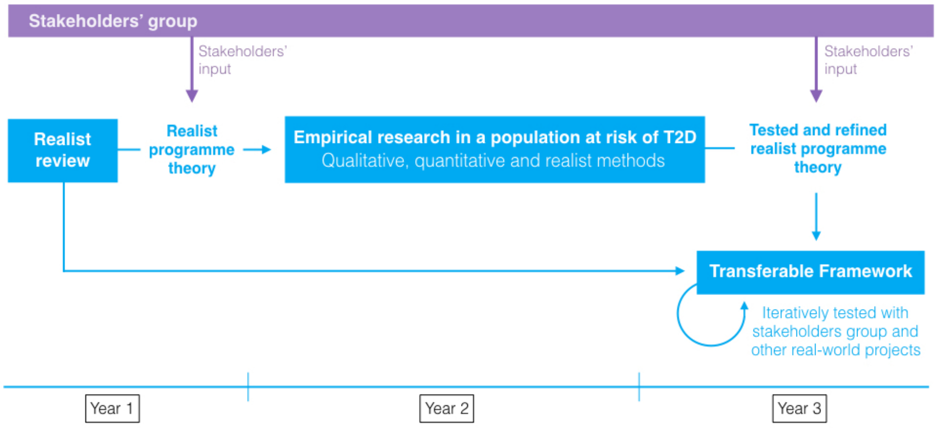


Figure 2. Overview of the study design

361x270mm (72 x 72 DPI)

Supplementary File 2. Literature searches

The final search strategy included combinations of search terms for the concepts 'social prescribing', 'primary care', 'community referral', 'diabetes', and was informed by previous related reviews on SP (1–3) and further developed in MEDLINE (Ovid) using an iterative process of adding, removing and refining search terms.

In September 2019, the following databases were searched: MEDLINE (Ovid), EMBASE (Ovid), PsycINFO (Ovid), Scopus (Elsevier), Web of Science (Clarivate Analytics), CINAHL Plus (EBSCO), PubMed (NCBI), International Bibliography of the Social Sciences - IBSS (ProQuest), The Cochrane Database of Systematic Reviews (The Cochrane Library), Campbell Collaboration, Open Grey (INISR-CNRS), King's Fund Database and LILACS (BIREME).

Database alerts were set up to identify studies published between October 2019 and March 2020.

**Database: MEDLINE**

Host: Ovid.

Date searched: 27 September 2019.

Searcher: Sara Calderón, Yasmin Milner.

Hits: 129.

Strategy:

1. (social prescri* and type 2 diabetes).mp.
2. (social prescri* or community referral).mp.
3. (social prescri* or community referral or non medical referral).mp.
4. (social prescri* and primary health care).mp.
5. (social prescri* and primary care).mp.
6. (social prescri* or community referral or referral scheme).mp.
7. ((((social prescri* or community referral) and primary care) or primary health care) and diabetes).mp.
8. ((social prescri* or community referral) and primary health care and diabetes).mp.
9. 1 or 2 or 3 or 4 or 5 or 8

**Database: EMBASE**

Host: Ovid.

Date searched: 27 September 2019.

Searcher: Sara Calderón, Yasmin Milner.

Hits: 271.

Strategy:

For peer review only

1. (social prescri* and type 2 diabetes).mp.
2. (social prescri* or community referral).mp.
3. (social prescri* or community referral or non medical referral).mp.
4. (social prescri* and primary health care).mp.
5. (social prescri* and primary care).mp.
6. (social prescri* or community referral or referral scheme).mp.
7. ((((social prescri* or community referral) and primary care) or primary health care) and diabetes).mp.
8. ((social prescri* or community referral) and primary health care and diabetes).mp.
9. 1 or 2 or 3 or 4 or 5 or 8

**Database: PsycINFO**

Host: Ovid.

Date searched: 27 September 2019.

Searcher: Sara Calderón, Yasmin Milner.

Hits: 113.

Strategy:

1. (social prescri* and type 2 diabetes).mp.
2. (social prescri* or community referral).mp.
3. (social prescri* or community referral or non medical referral).mp.
4. (social prescri* and primary health care).mp.
5. (social prescri* and primary care).mp.
6. (social prescri* or community referral or referral scheme).mp.
7. ((((social prescri* or community referral) and primary care) or primary health care) and diabetes).mp.
8. ((social prescri* or community referral) and primary health care and diabetes).mp.
9. 1 or 2 or 3 or 4 or 5 or 8

**Database: Scopus**

Host: Elsevier.

Date searched: 29 September 2019.

Searcher: Sara Calderón, Yasmin Milner.

Hits: 355.

Strategy:

1. TITLE-ABS-KEY ("Social Prescri*" OR "community referral" AND "type 2 diabetes")
2. TITLE-ABS-KEY ("Social Prescri*" OR "community referral")
3. TITLE-ABS-KEY ("Social Prescri*" OR "community referral" AND "diabetes" AND "primary health care")
4. TITLE-ABS-KEY ("Social Prescri*" OR "community referral" AND "primary health care")

For peer review only

5. TITLE-ABS-KEY ("Social Prescri*" AND "primary health care")

**Database: CINAHL Plus**

Host: EBSCO.

Date searched: 30 September 2019.

Searcher: Sara Calderón, Yasmin Milner.

Hits: 165.

Strategy:

1. "Social prescri*" OR "community referral"

**Database: PubMed**

Host: NCBI.

Date searched: 30 September 2019.

Searcher: Sara Calderón, Yasmin Milner.

Hits: 183.

Strategy:

1. ((((((social prescri*) OR "community referral")) AND "primary health care")) OR ((social prescri*) OR "community referral"))) OR ((((social prescri*) OR "community referral")) AND diabetes)
2. (((((social prescri*) OR "community referral")) AND "primary health care")) OR ((social prescri*) OR "community referral")

**Database: Web of Science**

Host: Clarivate Analytics.

Date searched: 30 September 2019.

Searcher: Sara Calderón, Yasmin Milner.

Hits: 183.

Strategy:

1. ("social prescri*")
2. ("social prescri*" OR "community referral")
3. ("social prescri*" OR "community referral") *AND* (diabetes)
4. ("social prescri*" OR "community referral") *AND* ("primary health care")
5. #4 OR #3 OR #2 OR #1

**Database: IBSS**

For peer review only

Host: ProQuest.

Date searched: 30 September 2019.

Searcher: Sara Calderón, Yasmin Milner.

Hits: 11.

Strategy:

1. noft("community referral") OR noft("social prescri*")

**Database: Cochrane Library**

Host: Cochrane Collaboration.

Date searched: 30 September 2019.

Searcher: Sara Calderón, Yasmin Milner.

Hits: 28.

Strategy:

1. "social prescri*" in Title Abstract Keyword OR "community referral" in Title Abstract Keyword AND "primary care" in Title Abstract Keyword
2. "social prescri*" in Title Abstract Keyword OR "community referral" in Title Abstract Keyword AND diabetes in Title Abstract Keyword
3. "social prescri*" in Title Abstract Keyword OR "community referral" in Title Abstract Keyword

**Database: Open Grey**

Host: INISR-CNRS.

Date searched: 30 September 2019.

Searcher: Sara Calderón, Yasmin Milner.

Hits: 1.

Strategy:

1. "social prescribing" OR "community referral"

**Database: LILACS**

Host: BIREME.

Date searched: 30 September 2019.

Searcher: Sara Calderón, Yasmin Milner.

For peer review only

Hits: 93.

Strategy:

1. (tw:("community referral"))
2. (tw:("community referral")) AND (tw:("social prescribing"))

**Database: King's Fund**

Host: The King's Fund.

Date searched: 3 October 2019.

Searcher: Sara Calderón, Yasmin Milner.

Hits: 218.

Strategy:

1. "social prescribing"
2. "community referral" AND "social prescribing"

REFERENCES:

1. Public Health England. Effectiveness of social prescribing - An evidence synthesis [Internet]. London; 2019 [cited 2019 Sep 29]. Available from: www.facebook.com/PublicHealthEngland

2. Pilkington K, Loef M, Polley M. Searching for Real-World Effectiveness of Health Care Innovations: Scoping Study of Social Prescribing for Diabetes. J Med Internet Res [Internet]. 2017 Feb 2 [cited 2018 May 14];19(2):e20–e20. Available from: http://www.ncbi.nlm.nih.gov/pubmed/28153817

3. Bickerdike L, Booth A, Wilson PM, Farley K, Wright K, Wilson PM. Social prescribing: less rhetoric and more reality. A systematic review of the evidence. BMJ Open [Internet]. 2017 [cited 2018 Jun 2];7(4):e013384. Available from: http://dx.doi.org/

For peer review only

# PRISMA-P 2015 Checklist

**This checklist has been adapted for use with protocol submissions to *Systematic Reviews* from Table 3 in Moher D et al:** Preferred reporting items for systematic review and meta-analysis protocols (PRISMA-P) 2015 statement. *Systematic Reviews* 2015 **4**:1

| Section/topic | # | Checklist item | Information reported | | Line number(s) |
|---|---|---|---|---|---|
| | | | Yes | No | |
| **ADMINISTRATIVE INFORMATION** | | | | | |
| **Title** | | | | | |
| Identification | 1a | Identify the report as a protocol of a systematic review | X | | P1, L3 |
| Update | 1b | If the protocol is for an update of a previous systematic review, identify as such | | | N/A |
| **Registration** | 2 | If registered, provide the name of the registry (e.g., PROSPERO) and registration number in the Abstract | X | | P2, L27 |
| **Authors** | | | | | |
| Contact | 3a | Provide name, institutional affiliation, and e-mail address of all protocol authors; provide physical mailing address of corresponding author | X | | P1, L5-15 |
| Contributions | 3b | Describe contributions of protocol authors and identify the guarantor of the review | X | | P16, L10 |
| **Amendments** | 4 | If the protocol represents an amendment of a previously completed or published protocol, identify as such and list changes; otherwise, state plan for documenting important protocol amendments | | | N/A |
| **Support** | | | | | |
| Sources | 5a | Indicate sources of financial or other support for the review | X | | P16, L13 |
| Sponsor | 5b | Provide name for the review funder and/or sponsor | X | | P16, L13 |
| Role of sponsor/funder | 5c | Describe roles of funder(s), sponsor(s), and/or institution(s), if any, in developing the protocol | X | | P16, L13 |
| **INTRODUCTION** | | | | | |
| **Rationale** | 6 | Describe the rationale for the review in the context of what is already known | X | | P4, L3-32 |
| **Objectives** | 7 | Provide an explicit statement of the question(s) the review will address with reference to participants, interventions, comparators, and outcomes (PICO) | X | | P6, L20-32 |
| **METHODS** | | | | | |
| **Eligibility criteria** | 8 | Specify the study characteristics (e.g., PICO, study design, setting, time frame) and report | X | | P9, L7-16 |

BioMed Central The Open Access Publisher

| Section/topic | # | Checklist item | Information reported Yes | No | Line number(s) |
|---|---|---|---|---|---|
| | | characteristics (e.g., years considered, language, publication status) to be used as criteria for eligibility for the review | | | |
| **Information sources** | 9 | Describe all intended information sources (e.g., electronic databases, contact with study authors, trial registers, or other grey literature sources) with planned dates of coverage | X | | P9, L1-6 |
| **Search strategy** | 10 | Present draft of search strategy to be used for at least one electronic database, including planned limits, such that it could be repeated | X | | P8, L28 |
| ***STUDY RECORDS*** | | | | | |
| Data management | 11a | Describe the mechanism(s) that will be used to manage records and data throughout the review | X | | P9, L1-16 |
| Selection process | 11b | State the process that will be used for selecting studies (e.g., two independent reviewers) through each phase of the review (i.e., screening, eligibility, and inclusion in meta-analysis) | X | | P9, L1-16 |
| Data collection process | 11c | Describe planned method of extracting data from reports (e.g., piloting forms, done independently, in duplicate), any processes for obtaining and confirming data from investigators | X | | P9, L18-27 |
| **Data items** | 12 | List and define all variables for which data will be sought (e.g., PICO items, funding sources), any pre-planned data assumptions and simplifications | X | | P9, L23-27 |
| **Outcomes and prioritization** | 13 | List and define all outcomes for which data will be sought, including prioritization of main and additional outcomes, with rationale | X | | P9, L23-32 |
| **Risk of bias in individual studies** | 14 | Describe anticipated methods for assessing risk of bias of individual studies, including whether this will be done at the outcome or study level, or both; state how this information will be used in data synthesis | X | | P9, L8-16 |
| ***DATA*** | | | | | |
| **Synthesis** | 15a | Describe criteria under which study data will be quantitatively synthesized | | | N/A |
| | 15b | If data are appropriate for quantitative synthesis, describe planned summary measures, methods of handling data, and methods of combining data from studies, including any planned exploration of consistency (e.g., $I^2$, Kendall's tau) | | | N/A |
| | 15c | Describe any proposed additional analyses (e.g., sensitivity or subgroup analyses, meta-regression) | | | N/A |
| | 15d | If quantitative synthesis is not appropriate, describe the type of summary planned | X | | P9, L21-32 P10, L1-3 |
| **Meta-bias(es)** | 16 | Specify any planned assessment of meta-bias(es) (e.g., publication bias across studies, selective reporting within studies) | | | N/A |
| **Confidence in cumulative evidence** | 17 | Describe how the strength of the body of evidence will be assessed (e.g., GRADE) | X | | P10, L1-3 |

BioMed Central The Open Access Publisher

BMJ Open

# Could social prescribing contribute to type 2 diabetes prevention in people at high risk? Protocol for a realist multi-level, mixed-methods review and evaluation.

| | |
|---|---|
| Journal: | *BMJ Open* |
| Manuscript ID | bmjopen-2020-042303.R2 |
| Article Type: | Protocol |
| Date Submitted by the Author: | 04-Mar-2021 |
| Complete List of Authors: | Calderón Larrañaga, Sara; Queen Mary University of London,<br>Clinch , Megan; Barts and the London School of Medicine and Dentistry, Institute of Population Health Sciences<br>Greenhalgh, Trisha; University of Oxford, Nuffield Department of Primary Care Health Sciences<br>Finer, Sarah; Barts and the London School of Medicine and Dentistry, Queen Mary University of London, Blizard Institute |
| <b>Primary Subject Heading</b>: | General practice / Family practice |
| Secondary Subject Heading: | Health services research, Qualitative research, Public health, Diabetes and endocrinology |
| Keywords: | PRIMARY CARE, SOCIAL MEDICINE, DIABETES & ENDOCRINOLOGY |
| | |

SCHOLARONE™
Manuscripts

*I, the Submitting Author has the right to grant and does grant on behalf of all authors of the Work (as defined in the below author licence), an exclusive licence and/or a non-exclusive licence for contributions from authors who are: i) UK Crown employees; ii) where BMJ has agreed a CC-BY licence shall apply, and/or iii) in accordance with the terms applicable for US Federal Government officers or employees acting as part of their official duties; on a worldwide, perpetual, irrevocable, royalty-free basis to BMJ Publishing Group Ltd ("BMJ") its licensees and where the relevant Journal is co-owned by BMJ to the co-owners of the Journal, to publish the Work in this journal and any other BMJ products and to exploit all rights, as set out in our licence.*

*The Submitting Author accepts and understands that any supply made under these terms is made by BMJ to the Submitting Author unless you are acting as an employee on behalf of your employer or a postgraduate student of an affiliated institution which is paying any applicable article publishing charge ("APC") for Open Access articles. Where the Submitting Author wishes to make the Work available on an Open Access basis (and intends to pay the relevant APC), the terms of reuse of such Open Access shall be governed by a Creative Commons licence – details of these licences and which Creative Commons licence will apply to this Work are set out in our licence referred to above.*

*Other than as permitted in any relevant BMJ Author's Self Archiving Policies, I confirm this Work has not been accepted for publication elsewhere, is not being considered for publication elsewhere and does not duplicate material already published. I confirm all authors consent to publication of this Work and authorise the granting of this licence.*

For peer review only

**TITLE:**

Could social prescribing contribute to type 2 diabetes prevention in people at high risk? Protocol for a realist multi-level, mixed-methods review and evaluation.

**CORRESPONDING AUTHOR:** Sara Calderón-Larrañaga, s.calderon@qmul.ac.uk. Postal address: 58 Turner St, Whitechapel, London E1 2AB.

**AUTHORS**: Sara Calderón-Larrañaga (1,2), Megan Clinch (1), Trisha Greenhalgh (3), Sarah Finer (1,4).

1. Centre for Primary Care and Mental Health, Institute of Population Health Sciences, Queen Mary University of London. London, UK
2. Bromley By Bow Health Partnership. London, UK
3. Nuffield Department of Primary Care Health Sciences, University of Oxford, Oxford, UK
4. Barts Health NHS Trust, London, UK

**WORD COUNT:** 3,611

**KEYWORDS:** social prescribing, type 2 diabetes, primary health care, realist methodology

For peer review only

**ABSTRACT:**

**Introduction.** Social Prescribing is an innovation being widely adopted within the UK National Health Service Policy as a way of improving the management of people with long-term conditions, such as type 2 diabetes (T2D). It generally involves linking patients in primary care with non-medical community-based interventions. Despite widespread national support, evidence for the effectiveness of social prescribing is both insufficient and contested. In this study, we will investigate whether social prescribing can contribute to T2D prevention and, if so, when, how and in what circumstances it might best be introduced.

**Methods and analysis.** We will draw on realist evaluation to investigate the complex interpersonal, organizational, social and policy context in which social prescribing relevant to T2D prevention is implemented. We will set up a stakeholders' group to advise us throughout the study, which will be conducted over 3 interconnected stages. In Stage 1, we will undertake a realist review to synthesize the current evidence base for social prescribing. In Stage 2, we will investigate how social prescribing relevant to people at high risk of T2D 'works' in a multi-ethnic, socio-economically diverse community and any interactions with existing T2D prevention services using qualitative, quantitative and realist methods. In Stage 3 and building on previous stages, we will synthesise a 'transferable framework' that will guide implementation and evaluation of social prescribing relevant to T2D prevention at scale.

**Ethics and Dissemination.** National Health Service ethics approval has been granted (reference 20/LO/0713). This project will potentially inform the adaptation of social prescribing services to better meet the needs of people at high risk of T2D in socioeconomically deprived areas. Findings may also be transferable to other long-term conditions. Dissemination will be undertaken as a continuous process, supported by the stakeholder group. Tailored outputs will target the following audiences: (1) service providers and commissioners; (2) people at high risk of T2D and community stakeholders; and (3) policy and strategic decision makers.

**Registration details.** PROSPERO (CRD42020196259)

For peer review only

**STRENGTHS AND LIMITATIONS OF THIS STUDY:**

- Our study contributes to a much-needed evidence base for both social prescribing (SP) and its real-world implementation in specific patient groups, such as those at high risk of type 2 diabetes (T2D)

- The realist methodology will enable to evaluate existing SP programmes within the wider relational, organisational and policy context where they are delivered, including any potential interactions with existing NHS T2D prevention services

- We will seek participatory involvement from our stakeholders' advisory group throughout the study, which will strengthen the practical relevance and transferability of findings

- The study does not intend to generate an effect size, but findings may guide the implementation and evaluation of SP initiatives relevant to T2D prevention at scale

For peer review only

**INTRODUCTION:**

Social Prescribing (SP) is an 'innovation' being widely adopted in the UK, including at policy-level within the National Health Service (NHS) 'Long Term Plan'(1,2). Although its definition varies, SP generally involves linking patients in primary care with community services offering employment, housing or financial advice, as well as a range of 'healthy lifestyle' activities, such as cooking classes, weight management or exercise programmes(3). Health and lifestyle issues are supposed to be addressed alongside socio-economic matters in an integrated way, usually with the help of a 'social prescriber' (also called 'link worker') who acts as link between health professionals and the wide range of community and voluntary services relevant to patients' situations(4,5). Activities are typically non-medicalised, provided locally and potentially more accessible and culturally appropriate to the local communities(6).

Proponents of SP suggest it may improve health outcomes and redress health inequalities by addressing patients' social and behavioural determinants of health(7). It is also argued that SP could improve the efficient use of NHS and social care resources by strengthening community networks and enhancing self-care(8). However, current evidence base for SP is both scarce and contested. A recent systematic review published by Public Health England comprising 8 UK studies (1 cluster RCT and 7 pre/post-test studies) found no clear evidence for the effectiveness of SP schemes(9). In an earlier systematic review, Bickerdike and colleagues did not find enough evidence to prove either success or value for money across 15 studies(10). Further systematic reviews on SP have failed to report consistent health, service utilization or cost results(11-15).

Authors suggest that the evidence gap on SP may be, in part, due to inadequate methodological approaches to gathering, assessing and/or synthesising data(3). Because SP interventions have fuzzy boundaries, multiple and inter-related components and are extremely sensitive to the context, conventional research designs (namely randomised controlled trials (RCTs) and/or previous systematic reviews) may not be the most appropriate approach to evaluation (16-18). Although RCTs would enable to compare the effectiveness of different variations of SP programmes, the methodological requirements to do so could impede the analysis of the interplay between programme implementation, the individuals involved, the programme's context and the wider social environment(19). Novel methodological approaches are increasingly being used to understand 'why', 'for whom', and 'in what circumstances' SP might (or might not) work(5,20,21). However, no previous studies have evaluated SP in the context of


For peer review only - http://bmjopen.bmj.com/site/about/guidelines.xhtml

complex interpersonal, organisational and policy relations, nor their influence on service delivery and implementation.

There is also a need to evaluate the role of SP in specific patient groups and populations(22,23). This study will use type 2 diabetes (T2D) prevention as an exemplar and investigate the impact of SP in people at high risk of the condition based on the following considerations. First, T2D is a major public health concern, associated with reduced quality of life and life expectancy, and considerable socioeconomic consequences(24). Although individual behavioural risk factors, such as physical inactivity and poor nutrition, are important determinants of T2D risk, these are heavily influenced and constrained by higher-level social and demographic factors(25,26), which appear to be major driving forces behind escalating T2D epidemics and health inequalities(27,28). For T2D prevention strategies to be effective at population level and in the most high-risk groups, they most likely will need to address these social determinants in addition to focusing purely on individual-level behavioural interventions(29). Second, current NHS delivered T2D prevention programmes, such as 'Healthier You'(30), have not demonstrated high uptake(31). The Healthier You intervention consists of 18 educational sessions over the course of 9 months offered to people at high risk of T2D, but its effectiveness in preventing the condition remains uncertain(32). Additionally, a social gradient exists in those engaging with, and gaining benefit from the Healthier You programme(33). There is significant concern that low uptake and high attrition may, at least in part, reflect the fact that individualised behavioural interventions do not adequately address critical social determinants of T2D(34, 35). Evaluating the impact of SP on a specific health area, such as T2D, will potentially enable to examine whether and how such interventions may be able to complement existing individualised behavioural approaches.

**Setting and context:**

This study will be based in Tower Hamlets, a multi-ethnic inner-city district in the East End of London, UK, where overall and T2D-related health outcomes have proved to be poorer compared to the national average(36) despite high quality care(37). Tower Hamlets is one of the most deprived boroughs in the UK and is characterised by its great ethnic diversity, with over half of all residents having a minority ethnic background(38).

Local health intelligence data suggests that age-adjusted deaths directly attributable to diabetes are 8.93 per 100,000, compared to 5.06 across London and 5.06 across England(37). Rates of T2D in Tower Hamlets are significantly higher amongst sub-populations of lowest socio-economic status and South-Asian population(36,38,39). For instance, South Asians have a risk



For peer review only - http://bmjopen.bmj.com/site/about/guidelines.xhtml

of developing T2D that is two-fold greater than the local white population(40) and suffer from faster chronic kidney progression when they have diabetes(41). Tower Hamlets has become one of the 27 areas across the country chosen to be part of the first wave rollout of the national NHS Diabetes Prevention Programme, 'Healthier You'.

Tower Hamlets is also at the forefront of another innovative service model, SP, that is being rapidly adopted throughout the NHS. Tower Hamlets has a long-established local voluntary and community sector (VCS)(42) who have a long history of delivering health promotion activities, including those relevant to T2D prevention. SP is being rolled out borough-wide by the Clinical Commissioning Group, aimed at strengthening and expanding the partnership between the health and voluntary sectors to all local Primary Health Care centres, building on the structure and experience developed in the pre-existing VCS and SP schemes(43). All patients registered with a Tower Hamlets GP, aged over 18 and expressing a 'non-clinical' support need are eligible for the local SP programme. Each of the borough's 36 primary care settings is linked to at least one 'link worker', whose role ranges from signposting to more intensive approaches involving patients' needs assessments, ongoing support and recommendations of relevant VCS services. (43).'

The proposed research will examine the existing evidence base for SP, and critically evaluate SP implementation and delivery in Tower Hamlets, using the following specific aims and questions:

**Aims:**

1. To investigate the possible impact of SP on people at high risk of T2D.
2. To inform the design, implementation and evaluation of SP initiatives relevant to people at high risk of T2D.

**Research questions:**

1. To what extent might SP meet the complex health and social needs of people at high risk of T2D in socio-economically diverse populations?
2. What does 'good' practice in SP relevant to people at high risk of T2D look like and what are the main conditions ('active ingredients') for achieving this?
3. How do existing SP programmes address questions of implementation and evaluation, and what are the knowledge gaps critical to understanding their success or failure within a health system?


For peer review only - http://bmjopen.bmj.com/site/about/guidelines.xhtml

**METHODS:**

**Theoretical and conceptual framework:**

We will draw on realism, a theory-driven methodology that seeks to facilitate deep understanding of how complex interventions, such as SP, work and in what circumstances(44, 45). A central aspect of realism is its analysis of causation, which rejects the standard Humean model of regular succession of events. Realism takes the view that 'what causes something to happen has nothing to do with the number of times we observe it happening, instead identifying causal (also called 'generative') mechanisms of action and explaining how they work, whether they have been activated, and if so under what conditions'(44). Our task as researchers will be to untangle the 'web of causation'(46) identified using this realist approach, and to critically illuminate the underlying structures and mechanisms that might shape and generate empirically observed outcomes.

In order to explore these underlying assumptions, realist methodology proposes the identification of context-mechanism-outcome configurations (CMOc)(47). A CMOc is a hypothesis that the programme works (or does not work) (O) because of the action of some underlying mechanisms (M), which only comes into operation in particular contexts (C). '*If* the right processes operate in the right conditions *then* the programme will prevail'(48). These kind of theoretical explanations, sufficiently abstract to draw transferable lessons but closed enough to the observable data to enable empirical testing and refinement, are referred to as 'programme theories'(49).

CMOc may link to each other over time, with the outcome of one phase becoming the context of the next configuration in the chain of implementation steps (known as 'ripple effect')(50). Yet, CMOc may also organize concentrically(48). In this study, we propose a layered and dynamic realist framework (Figure 1) to investigate the complex interpersonal, organisational, social and policy context in which SP relevant to T2D is implemented, and illuminate key domains and tensions at these different and interconnected levels. A theory-testing strategy will be used to unpack these causal mechanisms and interrelated CMOc. Preliminary theoretical explanations will be made explicit and iteratively tested, confronted and refined using relevant and varied evidence from the primary literature (realist review(51)) and/or empirical data (realist evaluation(52)).



For peer review only - http://bmjopen.bmj.com/site/about/guidelines.xhtml

**Research Plan:**

The study will be conducted in three interlinked stages over three years as follows:

- Stage 1 – Development and refinement of a realist programme theory for SP interventions relevant to people at high risk of T2D using a critical systematic literature review (realist synthesis).
- Stage 2 – Empirical testing and further refinement of a realist programme theory (guided by Stage 1) in a socio-economically deprived, ethnically-diverse community at high risk of T2D using qualitative, quantitative and realist methods (realist evaluation).
- Stage 3 – Synthesis of secondary (Stage 1) and primary (Stage 2) evidence, involving the development of a transferable framework for guiding implementation and evaluation of SP relevant to T2D prevention at scale.

In parallel and as represented in Figure 2, we will set up a stakeholder group comprising representatives of the VCS, primary care leaders, members of Public Health England and relevant national charitable organisations (such as SP Network, Deep End Group, and/or the National Academy for Social Prescribing). Via individual and group interviews, the study objectives and ongoing research outcomes will be iteratively shared, leading to discussions that will seek to explore and highlight stakeholders' opinions and concerns about SP, T2D prevention and the emerging programme theory.

**Stage 1. Development and refinement of a realist programme theory (Realist Synthesis)**

Following the Realist And Meta-narrative Evidence Syntheses: Evolving Standards (RAMESES)(51,53), we will undertake a realist review in four iterative stages: (A) Searching for evidence, (B) Selecting and appraising literature, (C) Extracting and organizing data and (D) Synthesizing the evidence and drawing conclusions(54).

(A) Searching for evidence

Searches will be organised and performed in two phases. The main search will be aimed at identifying the rationales for why and how SP might (or might not) play a role in T2D prevention and building preliminary programme theories that will be negotiated within the research and stakeholders' groups. We will then undertake targeted searches aimed at refining these candidate programme theories.



For peer review only

We will use free-text and indexing search terms employed in previous systematic reviews(9,10,22). Terms for the second search will depend on the results from the first phase and will be discussed with a specialist librarian and the review team. We will search for qualitative, quantitative and mixed-methods studies in relevant electronic databases and grey literature resources (the search strategy and databases for the main search are specified in Supplementary File 1). We will also undertake forwards and backwards citation chasing on included studies, hand-searching of key journals, and study author contact where appropriate(55). Results will be exported to Rayyan QCRI for de-deduplication. The searching process will be reproduced by a second reviewer for consistency.

(B) Selecting and appraising articles

Studies will be included if they consider adults (18+) in primary care settings and focus on interventions that link patients in primary care with community-based resources. Studies will be excluded if written in languages other than English, Spanish or French. Inclusion and exclusion criteria will continue to be refined throughout the review as theoretical understanding develops, and until theoretical saturation has been reached. Literature will be appraised based on their relevance at the point of inclusion ('does it contribute to theory building and testing?') and rigour ('are the methods used to generate the relevant data credible and trustworthy?')(53). A 10% random subsample of screening decisions will be reviewed by a second reviewer for consistency.

(C) Extracting and organizing data

The main reviewer will read all included articles, extract descriptive study characteristics and carry out manual coding. Full texts and the coding framework resulting from the manual analysis will then be uploaded into NVivo 11 (qualitative data management software). Initial coding will comprise broad conceptual categories that will progressively be refined both inductively and deductively, and reframed applying a realist logic of analysis. This means that we will work iteratively to identify sections of the text related to contexts (C), mechanisms (M) and outcomes (O) and organize data in level-specific CMOc(54). Again, a 10% random subsample of coded articles will be reviewed by a second reviewer for consistency, and disagreements will be solved by discussion.

(D) Synthesizing the evidence and drawing conclusions


For peer review only - http://bmjopen.bmj.com/site/about/guidelines.xhtml

We will use evidence synthesis processes proposed by realist methodology (e.g. juxtaposition of data sources, reconciling 'contradictory' or disconfirming data, consolidation of sources of evidence)(48) to articulate clear and contextualised explanations on how and why certain patterns related to SP relevant to T2D prevention occur. These theory-informed, evidence-based, multilevel propositions will guide the empirical realist evaluation and the development of a transferable framework in stages 2 and 3, respectively.

**Stage 2. Empirical testing and further refinement of a realist programme theory (Realist Evaluation)**

(A) Study Design

We will undertake a realist, multi-level and mixed-methods evaluation of an existing SP programme, based in Tower Hamlets, east London (the setting has been described above). The appropriateness and relevance of these approaches in the study and evaluation of complex, multi-dimensional, dynamic phenomena (such as SP) have been well stablished in the literature(56-58).

At microlevel, we will characterise SP users and investigate their experience throughout the SP pathway (from initial referral to subsequent assessment by the link worker and unfolding community-based activities), paying special attention to the dynamic interaction between the different actors involved (patients, primary care clinicians, link workers, members of local community organisations, local authority/public health services). At mesolevel, we will explore how a primary care service and the voluntary and community sector serving a community at high risk of T2D interact and operate to enable SP. We will also study the potential interaction of SP schemes with existing NHS T2D prevention programmes, such us 'Healthier You'. At macrolevel, we will investigate the barriers, facilitators and incentives to supporting SP relevant to T2D within the managerial and political sphere, and explore the interests and significances of SP for policymakers and other key stakeholders. Following a convergent mixed-methods study design, qualitative and quantitative data will be collected and analysed 'in parallel' (during a similar timeframe) using an interactive approach, where data collection and analysis might drive changes in ongoing data collection procedures(59,60).

(B) Data collection

For peer review only

Data will be generated through a number of qualitative and quantitative methods (Table 1):

- Individual and group interviews. We will purposively select SP users living in an area at high risk of T2D (including those who did not turn up for appointments or declined to engage with the activities), members of VCS organisations involved in SP, as well as local primary care clinicians, link workers and policy makers. Sampling will be guided by the strategies specified in table 1 and the findings of the realist review, as to the characteristics and circumstances that play a role in the success or failure of SP. Sample size will be determined by data saturation. An initial invitation letter and consent form will be provided and those wishing to participate will be contacted by a researcher. Interviews will be arranged at a time and location convenient to the participants, and bilingual health advocates will be used where necessary.
- Observations of SP community-based activities. The main researcher will observe a sample of SP community-based activities, paying special attention to the interplay between the individual experience ('emic') and the sociocultural context ('etic') where these activities unfold(61). Observational data on participants' behaviours, characteristics of activities, segments of dialogue between actors and contextual features will be kept in the form of field notes for ulterior analysis.
- Documents. Relevant local SP documentation and policy reports will be accessed for further analysis. We will ask each interviewed policymaker to reference key documents that might be guiding policy in this area.
- Quantitative individual-level data. We will characterise the clinical and socio-demographic features of SP users and the background population eligible for SP (including users of the local 'Healthier You' programme), using anonymised data from high quality primary care electronic health records (available from the Clinical Effectiveness Unit, Queen Mary University of London) and local authority registers.
- Quantitative service-level data. We will gather SP referrers' data by accessing primary care case management systems (EMIS) and/or referral forms. Quantifiable data of local public, private and non-profit institutions and citizens' associations will also be accessed using link worker's referral forms and the records of the organisations under study. We will collect SP costs data to test feasibility of a future scaled up evaluation of SP and T2D prevention frameworks and their potential impact on health systems.


For peer review only - http://bmjopen.bmj.com/site/about/guidelines.xhtml

**Table 1. Overview of data source and collection in Stage 2**

| Data source | Data collection |
|---|---|
| **SP service users** | Interviews:<br>• Approximately 2 focus groups and 7 in-depth interviews<br>• Purposive sampling, based on ethnicity, gender, age, diabetes risk, participation in SP |
| | Clinical data on all Tower Hamlets residents eligible for SP:<br>• Clinical measurements (HbA1c)<br>• Diagnosis codes (listed in QOF, including hypertension, diabetes, obesity)<br>• Disease risk (QRISK2, QDScore, prediabetes) |
| | Socio-demographic data on all Tower Hamlets residents eligible for SP:<br>• Age and gender<br>• Ethnicity<br>• Ethnic density of local geography (postcode)<br>• Country of birth<br>• First language spoken<br>• Socio-economic status (Index of Multiple Deprivation, by Lower-layer Super Output Area) |
| **Primary care professionals, including link workers** | Interviews:<br>• Approximately 2 focus groups and 7 in-depth interviews<br>• Purposive sampling based on professional profile, type of contract, demographic characteristics (age, gender, ethnicity) |
| | SP referrers' quantitative data:<br>• Demographic characteristics (age and gender)<br>• Primary care workers' professional profile and background<br>• Type of contract |
| **VCS organisations** | Interviews:<br>• Approximately 2 focus groups and 7 in-depth interviews<br>• Purposive sampling based on type of activities, community embeddedness |
| | Observations of SP activities |
| | Key documentation: protocols, referral templates, advertising, web pages, etc. |
| | SP providers' quantitative data:<br>• Characteristics of the VCS organisations: available resources, areas of interest<br>• Patients' attendance frequency |
| **SP service** | Overview of the SP service:<br>• Existing referral criteria and reasons for referral<br>• Number of referrals per network, surgery, health worker and profession. |
| | Health economic data, including:<br>• Staff contact<br>• Attendance rates<br>• Facilities use |
| **Policymakers and public health stakeholders** | Interviews:<br>• Approximately 5 semi-structured interviews<br>• Snowball sampling |
| | Key documents and policy reports on SP |

For peer review only

(C) Data analysis

Qualitative data will be analysed thematically to identify the patterns and theoretical explanations derived from its concepts, categories and interrelations(62). Data will be analysed according to particular features, but without losing sight of the 'big picture' and the contexts in which it arises(63). In combination with thematic analysis, we will apply a realist logic of analysis to the data to complement, confirm, confront and/or refine the programme theory deriving from the review. Data analysis and interpretation will be reflexively monitored(64). We will seek negative cases and triangulate within the research group to incorporate and maintain validity, and hence ensure rigour in the research process(65,66).

Quantitative data will be used to characterise the potential impact of SP on people at high risk of T2D, and in the context of other existing T2D prevention strategies. These data will not provide a definitive evaluation of SP, but will provide important context for the qualitative research findings and will indicate areas of focus and choice of endpoints (and their feasibility) for future at-scale evaluation. Data will be analysed using descriptive statistics to characterise the clinical and socio-demographic features of patients accessing SP. Comparative statistical tests will be used to look for differences in the SP recipient population compared to the total background population age 18+ (the eligibility criteria for SP is age 18+ and a subjective assessment of need), and to individuals participating in the NHS Type 2 Diabetes prevention programme, Healthier You. Multi-variate analysis will enable the identification of potential factors that might be associated with the likelihood of being referred into SP and characterise the uptake and equity of access to the programme across the borough. Considering that 2% of the population receives SP and 1 in 11 people in Tower Hamlets are at high risk of developing T2D (QDScore>20), we anticipate having sufficient power (0.8) to detect a 0.7% absolute difference (35% relative difference) in the likelihood of receiving SP amongst diabetes risk groups, using a type 1 error rate of 0.05.

(D) Synthesis and integration of data

Qualitative findings, framed within CMOCs, will be synthesised according micro, meso and macro levels (and interconnections between levels) that most accurately represent how and why SP programs relevant to T2D prevention might (or might not) 'work'. Quantitative outcomes will be used to provide deeper context to the multi-level program theory. We will undertake a narrative integration of findings(60) and use visual means(67) where appropriate.

For peer review only

**Stage 3. Development of a transferable framework to guide implementation and evaluation of SP relevant to T2D**

Building on the findings of the critical systematic literature review (Stage 1) and the empirical research (Stage 2), we will synthesise a transferable framework for understanding and evaluating SP programs relevant to people at high risk of T2D. In order to enhance generalisability, a near final version of the framework will be shared with stakeholders involved in SP programmes relevant to T2D nationwide and further refined based on their inputs.

Similar approaches, such as the NASSS Framework (for theorizing and evaluating Nonadoption, Abandonment, Scale-up, Spread, and Sustainability of health and care technologies)(68), have previously been successfully developed and used in the field of health services research and complex interventions, and have the potential to translate research findings to clinical care, reducing the 'research-implementation divide' that research on complex interventions frequently encounters.

**Ethics and dissemination**

HRA and Health and Care Research Wales (HCRW) ethics approval has been granted (reference 20/LO/0713). Standard rules apply for data security, confidentiality and information governance. We will seek informed consent for observations during community-based interventions and interviews. We will protect the confidentiality and privacy of participants in focus groups by requesting acceptance of a code of conduct to ensure that personal and private information is not shared outside the group.

Dissemination of study outputs will be undertaken as a continuous process and be facilitated by the stakeholders' group. We anticipate that the transferable framework developed in Stage 3 will constitute a key output. We will develop workshops, presentations and summary documents to make it accessible to potential users. Senior policy contacts, who are represented on our stakeholders' group, will be ideally placed to facilitate its wide-reaching dissemination and implementation. We plan outputs for four main audiences.

- For service providers (including primary care clinicians, link workers and the VCS), we will provide guidance on how to evaluate and improve local SP schemes.

For peer review only

- For people living in areas at high risk of T2D and community stakeholders, we will develop lay summaries and user-friendly versions of all our findings.

- For policy makers and strategic decision makers, we will produce succinct and accessible briefing papers of key findings aimed at informing prevailing policy and commissioning decisions.

- For the academic community, we will produce research publications in peer-reviewed, open access journals and conference presentations. The whole proposed research will constitute the core contents of a PhD thesis, whose submission and defence will be accomplished from the QMUL.

**Patient and public involvement**

Patient and Public Involvement (PPI) is central to the development of this project and for the plans on how it will be executed. To date, this involvement has come from several groups, including local east London community members (through pilot work to identify the research questions, scope and acceptability), the QMUL-Barts Health Diabetes Lay Research Panel, as well as using Diabetes UK resources including the recent James Lind Alliance T2D priority setting partnership (69). As previously explained, our 'stakeholders' advisory group' will also help to enhance the practical relevance and impact of our research.

## DISCUSSION

There is an increasing interest in SP as a means of addressing the wider social determinants behind escalating burden of long-term conditions, such as T2D, and health inequalities. As part of the NHS Long Term Plan(1), NHS England plans to recruit 1,000 SP link workers so that the service is available in every GP practice by 2024(70).

Despite widespread policy support and proliferation, evidence for the effectiveness of SP is currently sparse, its implementation is heterogeneous and complex, and it incorporates little robust evaluation across health domains(71). Some of the recent studies on SP have used novel methodological approaches to better understand 'how', 'why' and 'in what circumstances' it might or (might not) work(4,20,21). Qualitative research studies have also provided valuable insight into the processes of program implementation and delivery(72-75). However, no previous studies have addressed and evaluated SP in the context of dynamic, interconnected,


For peer review only - http://bmjopen.bmj.com/site/about/guidelines.xhtml

multilevel systems. Nor have they 'critically' analysed this context to purposively unfold the tensions, contradictions and competing interests that can potentially shape service delivery.

This study brings a novel methodological approach to investigate whether SP may contribute to comprehensive, community embedded T2D prevention strategies in primary care and its potential impact on patients at high risk. Our multilevel analytic approach will potentially illuminate the complexity of SP and the system in which it operates (including interpersonal, organisational, policy contexts). It is envisaged that project outcomes will inform the design, implementation and evaluation of SP initiatives relevant to T2D, by understanding when, how and in what circumstances this service model might best be introduced.

**Authors' contributions:** SCL and SF conceptualised the study with input from MC and TG. SCL wrote the first draft of this manuscript with significant input from SF, MC, and TG. All authors have read and approved the final manuscript.

**Funding:** This work is supported by the Economic Social Research Council, London Interdisciplinary Social Sciences Doctoral Training Partnership grant number ES/P000703/1 and Currier's Millennium Healthcare Bursary.

**Disclaimer:** The views and opinions expressed therein are those of the authors and do not necessarily reflect those of the London Interdisciplinary Social Sciences Doctoral Training Partnership, Currier's Millennium Healthcare Bursary, National Health Service or the Department of Health.

**Competing interests:** None declared

**Patient consent:** The submitted paper is a study protocol.

**Ethics approval:** Health Research Authority (HRA) and Health and Care Research Wales (HCRW) ethics approval (reference 20/LO/0713).

**Data sharing statement:** Given this is a study protocol, no data are available to be shared yet.

For peer review only

**REFERENCES:**


1. National Health Service (NHS). NHS Long Term Plan. London, UK; 2019. Available from: https://www.longtermplan.nhs.uk
2. Greenhalgh T, Robert G, Macfarlane F, et al. Diffusion of Innovations in Service Organizations: Systematic Review and Recommendations. Milbank Q. 2004;82(4):581–629.
3. Drinkwater C, Wildman J, Moffatt S. Social prescribing. BMJ. 2019 Mar 28;364:l1285.
4. Moffatt S, Steer M, Lawson S, et al. Link Worker social prescribing to improve health and well-being for people with long-term conditions: qualitative study of service user perceptions. BMJ Open. 2017;7(7):e015203–e015203.
5. Bertotti M, Frostick C, Hutt P, et al. A realist evaluation of social prescribing: an exploration into the context and mechanisms underpinning a pathway linking primary care with the voluntary sector. Prim Heal Care Res Dev. 2018;19(3):232–45.
6. University of York. Centre for Reviews and Dissemination. Evidence to inform the commissioning of social prescribing. 2015. Available from: https://www.york.ac.uk/media/crd/Ev%20briefing_social_prescribing.pdf
7. Social Prescribing Network. Report of the annual social prescribing network conference. London: University of Westminster; 2016.
8. Woodall J, Trigwell J, Bunyan A-M, et al. Understanding the effectiveness and mechanisms of a social prescribing service: a mixed method analysis. BMC Heal Serv Res. 2018;18(1):604.
9. Public Health England. Effectiveness of social prescribing - An evidence synthesis. London: PHE publications; 2019.
10. Bickerdike L, Booth A, Wilson PM, et al. Social prescribing: less rhetoric and more reality. A systematic review of the evidence. BMJ Open. 2017;7(4):e013384.
11. Gottlieb LM, Wing H, Adler NE. A Systematic Review of Interventions on Patients' Social and Economic Needs. Am J Prev Med. 2017 Nov;53(5):719–29.
12. Pescheny J V, Randhawa G, Pappas Y. The impact of social prescribing services on service users: a systematic review of the evidence. Eur j public Heal. 2019.
13. Polley M, Bertotti M, Kimberlee R, et al. A review of the evidence assessing impact of social prescribing on healthcare demand and cost implications. London: University of Westminster; 2017.
14. Pavey TG, Taylor AH, Fox KR, et al. Effect of exercise referral schemes in primary

care on physical activity and improving health outcomes: systematic review and meta-analysis. BMJ. 2011 Nov 4;343:d6462.

15. Chatterjee HJ, Camic PM, Lockyer B, et al. Non-clinical community interventions: a systematised review of social prescribing schemes. Arts Health. 2018 May 4;10(2):97–123.

16. Greenhalgh T, Papoutsi C. Studying complexity in health services research: desperately seeking an overdue paradigm shift. BMC Med. 2018 [cited 2018 Jun 22];16(95).

17. Petticrew M. Public health evaluation: epistemological challenges to evidence production and use. Evid Policy A J Res Debate Pract. 2013 Jan 28;9(1):87–95.

18. Craig P, Dieppe P, Macintyre S, Michie S, Nazareth I, Petticrew M. Developing and evaluating complex interventions: the new Medical Research Council guidance. BMJ. 2008 Sep 29;a1655.

19. Marchal B, Westhorp G, Wong G, et al. Realist RCTs of complex interventions – An oxymoron. Soc Sci Med. 2013 Oct;94:124–8.

20. Husk K, Blockley K, Lovell R, et al. What approaches to social prescribing work, for whom, and in what circumstances? A realist review. Heal Soc Care Community. 2019.

21. Tierney S, Wong G, Roberts N, et al. Supporting social prescribing in primary care by linking people to local assets: a realist review. BMC Med. 2020 Dec 13;18(1):49.

22. Pilkington K, Loef M, Polley M. Searching for Real-World Effectiveness of Health Care Innovations: Scoping Study of Social Prescribing for Diabetes. J Med Internet Res. 2017;19(2):e20–e20

23. Moffatt S, Wildman J, Pollard TM, et al. Evaluating the impact of a community-based social prescribing intervention on people with type 2 diabetes in North East England: Mixed-methods study protocol. BMJ Open. 2019;9(1).

24. NICE guideline [NG28]. Type 2 diabetes in adults: management. 2019

25. Coen SE. Connecting qualitative research on exercise and environment to public health agendas requires an equity lens. Health Place. 2018 Sep;53:264–7.

26. Darmon N, Drewnowski A. Does social class predict diet quality? Am J Clin Nutr. 2008 May 1;87(5):1107–17.

27. Espelt A, Arriola L, Borrell C, et al. Socioeconomic Position and Type 2 Diabetes Mellitus in Europe 1999- 2009: a Panorama of Inequalities. Curr Diabetes Rev. 2011 May 1;7(3):148–58.

28. Agardh E, Allebeck P, Hallqvist J, et al. Type 2 diabetes incidence and socio-

For peer review only

economic position: a systematic review and meta-analysis. Int J Epidemiol. 2011 Jun 1;40(3):804–18.

29. Unwin N, Whiting D, Roglic G. Social determinants of diabetes and challenges of prevention. Lancet. 2010 Jun;375(9733):2204–5.

30. England NHS. NHS Diabetes Prevention Programme (NHS DPP). London;2015. Available from: https://www.england.nhs.uk/ourwork/qual-clin-lead/diabetes-prevention/

31. Barry E, Roberts S, Oke J, et al. Efficacy and effectiveness of screen and treat policies in prevention of type 2 diabetes: systematic review and meta-analysis of screening tests and interventions. BMJ. 2017 Jan 4;i6538.

32. Davies MJ, Gray LJ, Troughton J, et al. A community based primary prevention programme for type 2 diabetes integrating identification and lifestyle intervention for prevention: the Let's Prevent Diabetes cluster randomised controlled trial. Prev Med (Baltim). 2016 Mar;84:48–56.

33. Valabhji J, Barron E, Bradley D, et al. Early Outcomes From the English National Health Service Diabetes Prevention Programme. Diabetes Care. 2020;43(1):152–60.

34. Barry E, Greenhalgh T, Fahy N. How are health-related behaviours influenced by a diagnosis of pre-diabetes? A meta-narrative review. BMC Med. 2018 Dec 27;16(1):121.

35. Barry E, Roberts S, Finer S, et al. Time to question the NHS diabetes prevention programme. BMJ. 2015 Sep 7;h4717.

36. Tower Hamlets Joint Strategic Needs Assessment Reference Group. Joint Strategic Needs Assessment. Summary Document. Life, Health and Wellbeing in Tower Hamlets. London, UK; 2016. Available from: https://www.towerhamlets.gov.uk/Documents/Public-Health/JSNA/JSNA_Summary.pdf

37. Tower Hamlets Joint Strategic Needs Assessment Reference Group. Joint Strategic Needs Assessment. Type 2 Diabetes 'Factsheet'. London, UK; 2015. Available from: https://www.towerhamlets.gov.uk/Documents/Public-Health/JSNA/Type_2_diabetes.pdf

38. Mole G. Health Equity in Primary Care in East London and the City: Data analysis to inform Joint Strategic Needs Assessment. London, UK; 2014. Available from: www.towerhamlets.gov.uk/Documents/Public-Health/TH-JSNA-Health-Equity-in-Primary-Care-2014.pdf

39. Noble D, Smith D, Mathur R, et al. Feasibility study of geospatial mapping of chronic

For peer review only

disease risk to inform public health commissioning. BMJ Open. 2012 Feb 15;2(1):e000711.

40. Mathur R, Noble D, Smith D, et al. Quantifying the risk of type 2 diabetes in East London using the QDScore: a cross-sectional analysis. Br J Gen Pract. 2012 Oct 1;62(603):e663–70.

41. Dreyer G, Hull S, Mathur R, et al. Progression of chronic kidney disease in a multi-ethnic community cohort of patients with diabetes mellitus. Diabet Med. 2013 Aug;30(8):956–63.

42. London Borough of Tower Hamlets. Voluntary and Community Sector Strategy 2016-2019. London, UK; 2016. Available from: https://www.towerhamlets.gov.uk/Documents/Strategy-and-performance/Draft_Voluntary_and_Community_Sector_Strategy_2016_2019.pdf

43. Ferguson K, Hogarth S. Social prescribing in Tower Hamlets: evaluation of borough-wide roll-out. London, UK; 2018. Available from: https://www.towerhamletstogether.com/resource-library

44. Sayer A. Realism and social science. London: Sage Publications; 2000.

45. Bhaskar R. A realist theory of science. 2nd ed. Brighton: Harvester Press; 1978.

46. Krieger N. Epidemiology and the web of causation: Has anyone seen the spider? Soc Sci Med. 1994 Oct;39(7):887–903.

47. Pawson R, Tiley N. Realistic evaluation. London: Sage; 1997.

48. Pawson R. The Science of Evaluation: A Realist Manifesto. London: Sage; 2013. 22–37 p.

49. Merton R. On Theoretical Sociology. Five Essays, Old and New. New York: The Free Press; 1967.

50. Jagosh J, Bush PL, Salsberg J, et al. A realist evaluation of community-based participatory research: partnership synergy, trust building and related ripple effects. BMC Public Health. 2015 Dec 30;15(1):725.

51. Wong G, Greenhalgh T, Westhorp G, et al. RAMESES publication standards: realist syntheses. BMC Med. 2013;11(21).

52. Wong G, Westhorp G, Manzano A, et al. RAMESES II reporting standards for realist evaluations. BMC Med. 2016;14(1):96.

53. Wong G, Greenhalgh T, Westhorp G, et al. Development of methodological guidance, publication standards and training materials for realist and meta-narrative reviews: the RAMESES (Realist And Meta-narrative Evidence Syntheses – Evolving Standards) project. Heal Serv Deliv Res. 2014;2:1–252.

For peer review only

54. Pawson R, Greenhalgh T, Harvey G WK. Realist Synthesis : An Introduction Realist. ESRC research methods programme Manchester: University of Manchester. 2014.
55. Greenhalgh T, Peacock R. Effectiveness and efficiency of search methods in systematic reviews of complex evidence: audit of primary sources. BMJ. 2005 Nov 5;331(7524):1064–5.
56. Maxwell J, Mittapalli K. Realism as a Stance for Mixed Methods Research. In: Tashakkori A, Teddlie C, editors. Handbook of Mixed Methods in Social and Behavioral Research. 2nd edition. Thousand Oaks, CA: Sage; 2010. p. 145–67.
57. Headley MG, Plano Clark VL. Multilevel Mixed Methods Research Designs: Advancing a Refined Definition. J Mix Methods Res. 2019 May 4;155868981984441.
58. Tariq S, Woodman J. Using mixed methods in health research. JRSM Short Rep. 2013;4(6): 2042533313479197.
59. Creswell JW, Plano Clark VL. Designing and Conducting Mixed Methods Research. Thousand Oaks, CA: Sage; 2007.
60. Fetters MD, Curry LA, Creswell JW. Achieving Integration in Mixed Methods Designs-Principles and Practices. Health Serv Res. 2013 Dec;48(6pt2):2134–56.
61. Hammersley M, Atkinson P. Ethnography: Principles in Practice. Third ed. London: Routledge; 2007.
62. Green J, Thorogood N. Qualitative methods for health research. 2nd ed. London: Sage; 2009.
63. Silverman D. Interpreting Qualitative Data. A Guide to the Principles of Qualitative Research. 4th ed. London: Sage; 2011.
64. Finlay L. 'Rigour', 'Ethical Integrity' or 'Artistry'? Reflexively Reviewing Criteria for Evaluating Qualitative Research. Br J Occup Ther. 2006 Jul 5;69(7):319–26.
65. Calderón C. Assessing the Quality of Qualitative Health Research: Criteria, Process and Writing. Forum Qual Soc Res. 2009;10(2:Art 17.).
66. Crabtree B, Miller W. Doing Qualitative Research. Thousand Oaks, CA: Sage; 2000.
67. Plano Clark V, Sanders K. The use of visual displays in mixed methods research: Strategies for effectively integrating the quantitative and qualitative components of a study. In: McCrudden M, Schaw G, Buckendahl C, editors. Use of visual displays in research and testing: Coding, interpreting, and reporting data. Charlotte, NC: Information Age Publishing; 2015. p. 177–206.
68. Greenhalgh T, Wherton J, Papoutsi C, et al. Beyond Adoption: A New Framework for Theorizing and Evaluating Nonadoption, Abandonment, and Challenges to the Scale-Up, Spread, and Sustainability of Health and Care Technologies. J Med

For peer review only

Internet Res. 2017 Nov 1;19(11):e367.

69. Finer S, Robb P, Cowan K, et al. Setting the top 10 research priorities to improve the health of people with Type 2 diabetes: a Diabetes UK-James Lind Alliance Priority Setting Partnership. Diabet Med. 2018;35(7):862–70.

70. Hancock M. Keynote Address. Paper presented at the Social prescribing: Coming of age. London: King's Fund. 2018. Available from: https://www.kingsfund.org.uk/events/social-prescribing

71. Husk K, Elston J, Gradinger F, et al. Social prescribing: where is the evidence? Br J Gen Pr. 2019;69(678):6–7.

72. South J, Higgins TJ, Woodall J, et al. Can social prescribing provide the missing link? Prim Heal Care Res Dev. 2008 Oct 4;9(4):310–8.

73. White JM, Cornish F, Kerr S. Front-line perspectives on 'joined-up' working relationships: a qualitative study of social prescribing in the west of Scotland. Heal Soc Care Community. 2017;25(1):194-203.

74. Skivington K, Smith M, Chng NR, et al. Delivering a primary care-based social prescribing initiative: a qualitative study of the benefits and challenges. Br J Gen Pr. 2018;68(672):e487–94.

75. Wildman JM, Moffatt S, Steer M, et al. Service-users' perspectives of link worker social prescribing: a qualitative follow-up study. BMC Public Health. 2019;19(1):98.

For peer review only

**FIGURE LEGEND**

**Figure 1.** Multi-level and dynamic realist framework

**Figure 2.** Overview of the study design

For peer review only

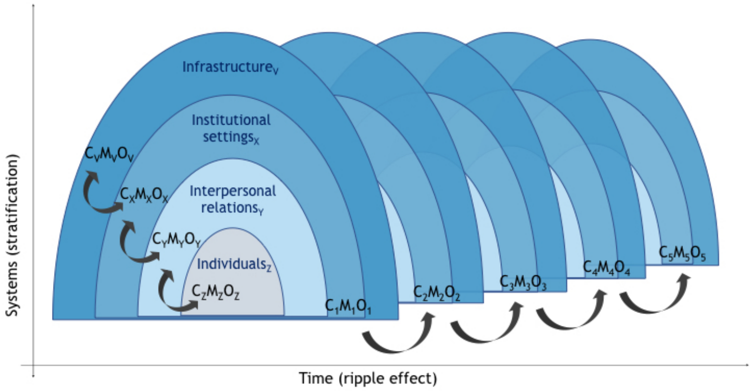


Figure 1. Multi-level realist framework

338x190mm (72 x 72 DPI)

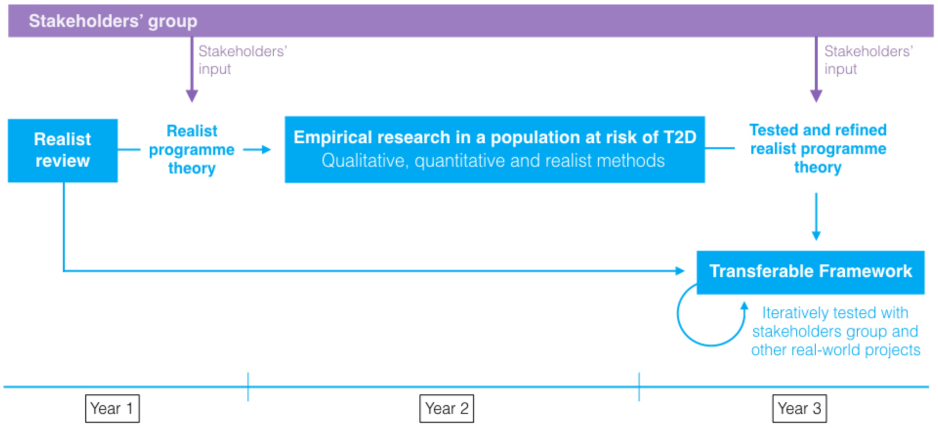


Figure 2. Overview of the study design

361x270mm (72 x 72 DPI)

Supplementary File 1. Literature searches

The final search strategy included combinations of search terms for the concepts 'social prescribing', 'primary care', 'community referral', 'diabetes', and was informed by previous related reviews on SP (1–3) and further developed in MEDLINE (Ovid) using an iterative process of adding, removing and refining search terms.

In September 2019, the following databases were searched: MEDLINE (Ovid), EMBASE (Ovid), PsycINFO (Ovid), Scopus (Elsevier), Web of Science (Clarivate Analytics), CINAHL Plus (EBSCO), PubMed (NCBI), International Bibliography of the Social Sciences - IBSS (ProQuest), The Cochrane Database of Systematic Reviews (The Cochrane Library), Campbell Collaboration, Open Grey (INISR-CNRS), King's Fund Database and LILACS (BIREME).

Database alerts were set up to identify studies published between October 2019 and March 2020.

**Database: MEDLINE**

Host: Ovid.

Date searched: 27 September 2019.

Searcher: Sara Calderón, Yasmin Milner.

Hits: 129.

Strategy:

1. (social prescri* and type 2 diabetes).mp.
2. (social prescri* or community referral).mp.
3. (social prescri* or community referral or non medical referral).mp.
4. (social prescri* and primary health care).mp.
5. (social prescri* and primary care).mp.
6. (social prescri* or community referral or referral scheme).mp.
7. ((((social prescri* or community referral) and primary care) or primary health care) and diabetes).mp.
8. ((social prescri* or community referral) and primary health care and diabetes).mp.
9. 1 or 2 or 3 or 4 or 5 or 8

**Database: EMBASE**

Host: Ovid.

Date searched: 27 September 2019.

Searcher: Sara Calderón, Yasmin Milner.

Hits: 271.

Strategy:

For peer review only

1. (social prescri* and type 2 diabetes).mp.
2. (social prescri* or community referral).mp.
3. (social prescri* or community referral or non medical referral).mp.
4. (social prescri* and primary health care).mp.
5. (social prescri* and primary care).mp.
6. (social prescri* or community referral or referral scheme).mp.
7. ((((social prescri* or community referral) and primary care) or primary health care) and diabetes).mp.
8. ((social prescri* or community referral) and primary health care and diabetes).mp.
9. 1 or 2 or 3 or 4 or 5 or 8

**Database: PsycINFO**

Host: Ovid.

Date searched: 27 September 2019.

Searcher: Sara Calderón, Yasmin Milner.

Hits: 113.

Strategy:

1. (social prescri* and type 2 diabetes).mp.
2. (social prescri* or community referral).mp.
3. (social prescri* or community referral or non medical referral).mp.
4. (social prescri* and primary health care).mp.
5. (social prescri* and primary care).mp.
6. (social prescri* or community referral or referral scheme).mp.
7. ((((social prescri* or community referral) and primary care) or primary health care) and diabetes).mp.
8. ((social prescri* or community referral) and primary health care and diabetes).mp.
9. 1 or 2 or 3 or 4 or 5 or 8

**Database: Scopus**

Host: Elsevier.

Date searched: 29 September 2019.

Searcher: Sara Calderón, Yasmin Milner.

Hits: 355.

Strategy:

1. TITLE-ABS-KEY ("Social Prescri*" OR "community referral" AND "type 2 diabetes")
2. TITLE-ABS-KEY ("Social Prescri*" OR "community referral")
3. TITLE-ABS-KEY ("Social Prescri*" OR "community referral" AND "diabetes" AND "primary health care")
4. TITLE-ABS-KEY ("Social Prescri*" OR "community referral" AND "primary health care")

For peer review only

5. TITLE-ABS-KEY ("Social Prescri*" AND "primary health care")

**Database: CINAHL Plus**

Host: EBSCO.

Date searched: 30 September 2019.

Searcher: Sara Calderón, Yasmin Milner.

Hits: 165.

Strategy:

1. "Social prescri*" OR "community referral"

**Database: PubMed**

Host: NCBI.

Date searched: 30 September 2019.

Searcher: Sara Calderón, Yasmin Milner.

Hits: 183.

Strategy:

1. ((((((social prescri*) OR "community referral")) AND "primary health care")) OR ((social prescri*) OR "community referral"))) OR ((((social prescri*) OR "community referral")) AND diabetes)
2. (((((social prescri*) OR "community referral")) AND "primary health care")) OR ((social prescri*) OR "community referral")

**Database: Web of Science**

Host: Clarivate Analytics.

Date searched: 30 September 2019.

Searcher: Sara Calderón, Yasmin Milner.

Hits: 183.

Strategy:

1. ("social prescri*")
2. ("social prescri*" OR "community referral")
3. ("social prescri*" OR "community referral") *AND* (diabetes)
4. ("social prescri*" OR "community referral") *AND* ("primary health care")
5. #4 OR #3 OR #2 OR #1

**Database: IBSS**

For peer review only

Host: ProQuest.

Date searched: 30 September 2019.

Searcher: Sara Calderón, Yasmin Milner.

Hits: 11.

Strategy:

1. noft("community referral") OR noft("social prescri*")

**Database: Cochrane Library**

Host: Cochrane Collaboration.

Date searched: 30 September 2019.

Searcher: Sara Calderón, Yasmin Milner.

Hits: 28.

Strategy:

1. "social prescri*" in Title Abstract Keyword OR "community referral" in Title Abstract Keyword AND "primary care" in Title Abstract Keyword
2. "social prescri*" in Title Abstract Keyword OR "community referral" in Title Abstract Keyword AND diabetes in Title Abstract Keyword
3. "social prescri*" in Title Abstract Keyword OR "community referral" in Title Abstract Keyword

**Database: Open Grey**

Host: INISR-CNRS.

Date searched: 30 September 2019.

Searcher: Sara Calderón, Yasmin Milner.

Hits: 1.

Strategy:

1. "social prescribing" OR "community referral"

**Database: LILACS**

Host: BIREME.

Date searched: 30 September 2019.

Searcher: Sara Calderón, Yasmin Milner.

For peer review only

Hits: 93.

Strategy:

1. (tw:("community referral"))
2. (tw:("community referral")) AND (tw:("social prescribing"))

**Database: King's Fund**

Host: The King's Fund.

Date searched: 3 October 2019.

Searcher: Sara Calderón, Yasmin Milner.

Hits: 218.

Strategy:

1. "social prescribing"
2. "community referral" AND "social prescribing"

REFERENCES:

1. Public Health England. Effectiveness of social prescribing - An evidence synthesis [Internet]. London; 2019 [cited 2019 Sep 29]. Available from: www.facebook.com/PublicHealthEngland

2. Pilkington K, Loef M, Polley M. Searching for Real-World Effectiveness of Health Care Innovations: Scoping Study of Social Prescribing for Diabetes. J Med Internet Res [Internet]. 2017 Feb 2 [cited 2018 May 14];19(2):e20–e20. Available from: http://www.ncbi.nlm.nih.gov/pubmed/28153817

3. Bickerdike L, Booth A, Wilson PM, Farley K, Wright K, Wilson PM. Social prescribing: less rhetoric and more reality. A systematic review of the evidence. BMJ Open [Internet]. 2017 [cited 2018 Jun 2];7(4):e013384. Available from: http://dx.doi.org/

For peer review only

1

# PRISMA-P 2015 Checklist

**This checklist has been adapted for use with protocol submissions to *Systematic Reviews* from Table 3 in Moher D et al:** Preferred reporting items for systematic review and meta-analysis protocols (PRISMA-P) 2015 statement. *Systematic Reviews* 2015 **4**:1

| Section/topic | # | Checklist item | Information reported Yes | No | Line number(s) |
|---|---|---|---|---|---|
| **ADMINISTRATIVE INFORMATION** | | | | | |
| **Title** | | | | | |
| Identification | 1a | Identify the report as a protocol of a systematic review | X | | P1, L3 |
| Update | 1b | If the protocol is for an update of a previous systematic review, identify as such | | | N/A |
| **Registration** | 2 | If registered, provide the name of the registry (e.g., PROSPERO) and registration number in the Abstract | X | | P2, L27 |
| **Authors** | | | | | |
| Contact | 3a | Provide name, institutional affiliation, and e-mail address of all protocol authors; provide physical mailing address of corresponding author | X | | P1, L5-15 |
| Contributions | 3b | Describe contributions of protocol authors and identify the guarantor of the review | X | | P16, L10 |
| **Amendments** | 4 | If the protocol represents an amendment of a previously completed or published protocol, identify as such and list changes; otherwise, state plan for documenting important protocol amendments | | | N/A |
| **Support** | | | | | |
| Sources | 5a | Indicate sources of financial or other support for the review | X | | P16, L13 |
| Sponsor | 5b | Provide name for the review funder and/or sponsor | X | | P16, L13 |
| Role of sponsor/funder | 5c | Describe roles of funder(s), sponsor(s), and/or institution(s), if any, in developing the protocol | X | | P16, L13 |
| **INTRODUCTION** | | | | | |
| **Rationale** | 6 | Describe the rationale for the review in the context of what is already known | X | | P4, L3-32 |
| **Objectives** | 7 | Provide an explicit statement of the question(s) the review will address with reference to participants, interventions, comparators, and outcomes (PICO) | X | | P6, L20-32 |
| **METHODS** | | | | | |
| **Eligibility criteria** | 8 | Specify the study characteristics (e.g., PICO, study design, setting, time frame) and report | X | | P9, L7-16 |

BioMed Central The Open Access Publisher

| Section/topic | # | Checklist item | Information reported Yes | No | Line number(s) |
|---|---|---|---|---|---|
| | | characteristics (e.g., years considered, language, publication status) to be used as criteria for eligibility for the review | | | |
| **Information sources** | 9 | Describe all intended information sources (e.g., electronic databases, contact with study authors, trial registers, or other grey literature sources) with planned dates of coverage | X | | P9, L1-6 |
| **Search strategy** | 10 | Present draft of search strategy to be used for at least one electronic database, including planned limits, such that it could be repeated | X | | P8, L28 |
| ***STUDY RECORDS*** | | | | | |
| Data management | 11a | Describe the mechanism(s) that will be used to manage records and data throughout the review | X | | P9, L1-16 |
| Selection process | 11b | State the process that will be used for selecting studies (e.g., two independent reviewers) through each phase of the review (i.e., screening, eligibility, and inclusion in meta-analysis) | X | | P9, L1-16 |
| Data collection process | 11c | Describe planned method of extracting data from reports (e.g., piloting forms, done independently, in duplicate), any processes for obtaining and confirming data from investigators | X | | P9, L18-27 |
| **Data items** | 12 | List and define all variables for which data will be sought (e.g., PICO items, funding sources), any pre-planned data assumptions and simplifications | X | | P9, L23-27 |
| **Outcomes and prioritization** | 13 | List and define all outcomes for which data will be sought, including prioritization of main and additional outcomes, with rationale | X | | P9, L23-32 |
| **Risk of bias in individual studies** | 14 | Describe anticipated methods for assessing risk of bias of individual studies, including whether this will be done at the outcome or study level, or both; state how this information will be used in data synthesis | X | | P9, L8-16 |
| ***DATA*** | | | | | |
| **Synthesis** | 15a | Describe criteria under which study data will be quantitatively synthesized | | | N/A |
| | 15b | If data are appropriate for quantitative synthesis, describe planned summary measures, methods of handling data, and methods of combining data from studies, including any planned exploration of consistency (e.g., $I^2$, Kendall's tau) | | | N/A |
| | 15c | Describe any proposed additional analyses (e.g., sensitivity or subgroup analyses, meta-regression) | | | N/A |
| | 15d | If quantitative synthesis is not appropriate, describe the type of summary planned | X | | P9, L21-32<br>P10, L1-3 |
| **Meta-bias(es)** | 16 | Specify any planned assessment of meta-bias(es) (e.g., publication bias across studies, selective reporting within studies) | | | N/A |
| **Confidence in cumulative evidence** | 17 | Describe how the strength of the body of evidence will be assessed (e.g., GRADE) | X | | P10, L1-3 |

For peer review only



BioMed Central
The Open Access Publisher
